# Follicular Helper T Cells Promote Liver Pathology in Mice during *Schistosoma japonicum* Infection

**DOI:** 10.1371/journal.ppat.1004097

**Published:** 2014-05-01

**Authors:** Xiaojun Chen, Xiaowei Yang, Yong Li, Jifeng Zhu, Sha Zhou, Zhipeng Xu, Lei He, Xue Xue, Weiwei Zhang, Xiaoxiao Dong, Henry Wu, Carrie J. Li, Hsiang-Ting Hsu, Wenjun Kong, Feng Liu, Prem B. Tripathi, Michelle S. Yu, Jason Chang, Liang Zhou, Chuan Su

**Affiliations:** 1 Department of Pathogen Biology & Immunology, Jiangsu Key Laboratory of Pathogen Biology, Nanjing Medical University, Nanjing, Jiangsu, P. R. China; 2 Department of Pathology, Department of Microbiology and Immunology, Feinberg School of Medicine, Northwestern University, Chicago, Illinois, United States of America; 3 Department of Pediatrics, Children's Hospital Los Angeles, Los Angeles, California, United States of America; 4 Keck School of Medicine of University of Southern California, Los Angeles, California, United States of America; 5 Department of General Surgery, Kaiser LAMC, Los Angeles, California, United States of America; University of Medicine & Denistry New Jersey, United States of America

## Abstract

Following *Schistosoma japonicum* (*S. japonicum*) infection, granulomatous responses are induced by parasite eggs trapped in host organs, particular in the liver, during the acute stage of disease. While excessive liver granulomatous responses can lead to more severe fibrosis and circulatory impairment in chronically infected host. However, the exact mechanism of hepatic granuloma formation has remained obscure. In this study, we for the first time showed that follicular helper T (Tfh) cells are recruited to the liver to upregulate hepatic granuloma formation and liver injury in *S. japonicum*-infected mice, and identified a novel function of macrophages in Tfh cell induction. In addition, our results showed that the generation of Tfh cells driven by macrophages is dependent on cell–cell contact and the level of inducible costimulator ligand (ICOSL) on macrophages which is regulated by CD40–CD40L signaling. Our findings uncovered a previously unappreciated role for Tfh cells in liver pathology caused by *S. japonicum* infection in mice.

## Author Summary

Schistosomiasis is a chronic helminthic disease that affects approximately 200 million people. After *S. japonicum* infection, parasite eggs are trapped in host liver and granulomas are induced to form around eggs. Severe granuloma subsequently results in serious liver fibrosis and circulatory impairment chronically. It is important to fully elucidate the mechanism of the granuloma formation. Here, we show that Tfh cells play a novel role of promoting the hepatic granuloma formation and liver injury, and identified a novel function of macrophages in Tfh cells induction in *S. japonicum*-infected mouse model. In addition, we show that the generation of Tfh cells driven by macrophages is cell–cell contact dependent and regulated by CD40-CD40L signaling. Our findings revealed a novel role and mechanism of macrophages in Tfh cell generation and the liver pathogenesis in *S. japonicum*-infected mouse model.

## Introduction

Schistosomiasis remains a major public health problem in many developing countries in tropical and subtropical regions, which affects approximately 200 million people worldwide [1], [2]. Following *Schistosoma japonicum* and *mansoni* (*S. japonicum* and *mansoni*) infections, eggs are trapped in host liver and trigger the formation of granuloma to surround eggs. However, severer granulomatous response precipitates fibrosis in the liver more quickly and eventually cause extensive tissue scarring, which in turn causes severer circulatory impairment of the affected organs [3]. Thus, better understanding the mechanism of granuloma formation is crucial to prevent excessive granulomatous lesions in schistosome infection.

Through an unclear mechanism, CD4^+^ T cell response induced by egg antigens orchestrates the development of granulomatous lesions around individual eggs in host liver [3]. Naïve CD4^+^ helper T (Th) cells recognize schistosome egg antigens presented by antigen-presenting cells (APCs) to differentiate into distinct effector subsets. These include Th1, Th2, and Th17 cells or regulatory T (Treg) cells, which differentiate under specific cytokine milieu. These populations express chemokine receptors for their homing to liver and produce distinct profiles of effector cytokines, which play roles in liver granuloma formation and regulation. For example, Th2 and Th17 cells were reported to upregulate hepatic granuloma formation by secreting IL-4 and IL-17 respectively [4]–[6], while Th1 and Treg cells were reported to downregulate hepatic granuloma formation [5], [7].

Follicular helper T (Tfh) cells are a further distinguishable subset of Th cells [8], [9], which are characterized by the expression of numerous molecules including the surface markers CXCR5, PD-1, and ICOS, and the transcription factor Bcl-6 [10], [11]. The reported key function of Tfh cells is to provide help to B cells to support their activation, expansion, and differentiation, as well as the formation of the germinal center (GC) [12], [13]. Recently, some evidence has emerged to support the role of Tfh cells in the development of autoimmune pathology by providing help to B cells to produce autoantibodies [14]–[16]. Except for providing help to B cells, it is currently unknown whether Tfh cells have other functions. For example, previous reports showed that Tfh cells are induced and are the predominant source of IL-4 in reactive lymph nodes during helminth infection [17], [18]. Although Tfh cells were induced during schistosome infection [19] and most IL-4-producing CD4^+^ T cells in reactive lymph nodes produced in response to *S. mansoni* antigens are Tfh cells [20], it is not yet clear whether Tfh cells are involved in the development of liver pathology during schistosome infection.

A number of cellular interactions between antigen-presenting cells (APC) and naïve precursors underlie Tfh cell development. For example, B cells are important for the generation of Tfh cells [13], [21]–[25]. Dendritic cells (DCs) have been shown that can also drive Tfh cell development even in the absence of T-B cell interactions [26], [27]. In addition, late activator antigen-presenting cell [28] and plasma cells [29] are also reported to be involved in the generation of Tfh cells. However, little is known with regard to whether macrophages, one important subset of APCs and playing a key role in the liver granuloma formation in chronic schistosomiasis japonica [30], [31], are involved in the generation of Tfh cells.

In this study, we identified a novel role for Tfh cells in liver pathology by using a *S. japonicum*-infected mouse model, and provided new insights into the promotion of Tfh cell generation by macrophages.

## Results

### *S. japonicum* infection drives Tfh-cell generation

**Figure 1:**
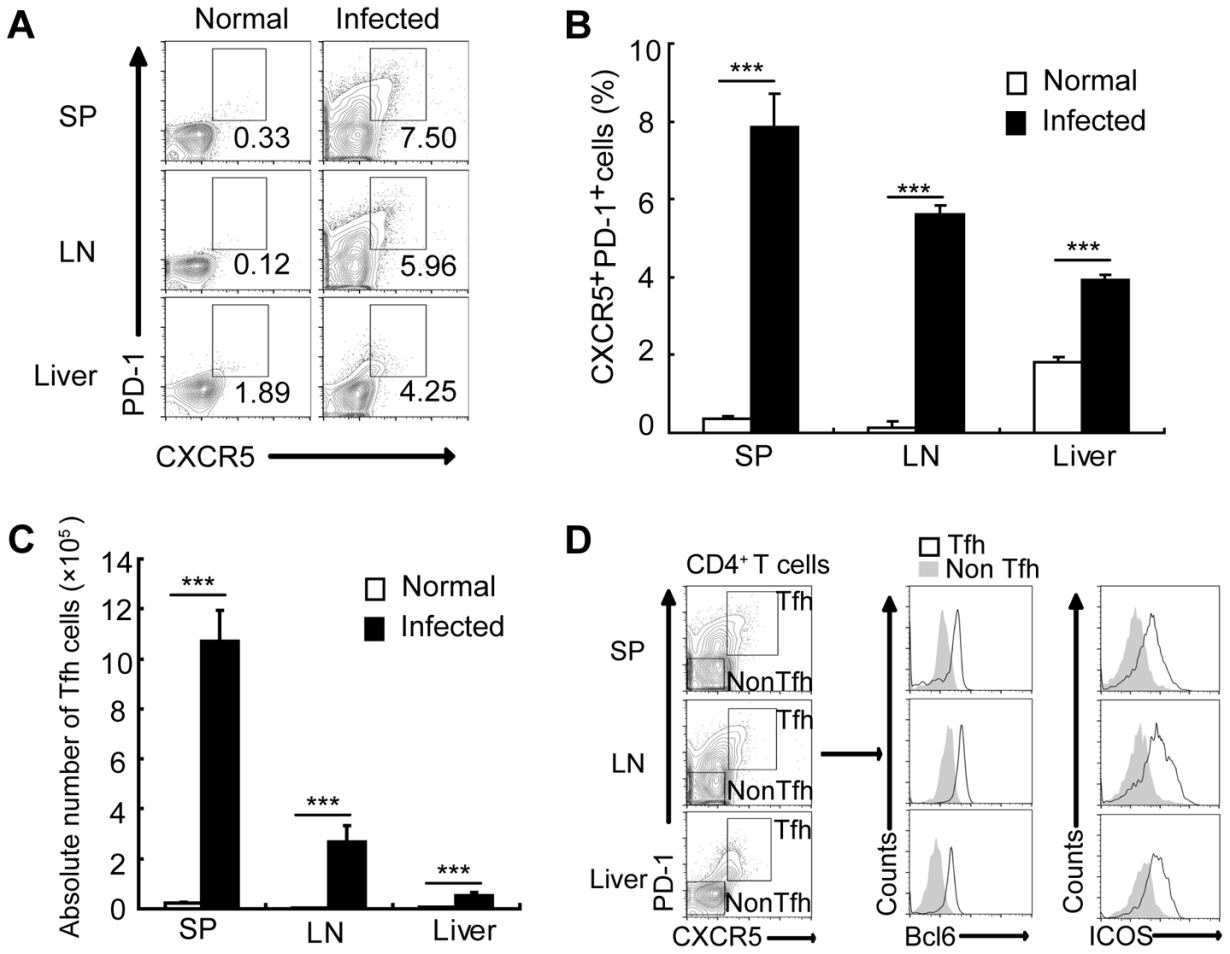
*S. japonicum* infection drives Tfh cell generation. For each of the three independent experiments, six male C57BL/6 mice were infected with 12 cercariae of *S. japonicum* per mouse. Infected mice were sacrificed at 8 weeks post-infection. (A) Spleens, mesenteric LN, and livers from normal and infected mice were harvested, and cells were stained with CD3-percpcy5.5, CD4-FITC, CXCR5-APC, and PD-1-PE antibodies. CXCR5^high^PD-1^high^ cells were analyzed and data shown are gated on CD4^+^ T cells. Numbers represent the frequency of the boxed population within the CD4^+^ T cell population; (B) Data are expressed as the mean ± SD of 12 mice from three independent experiments, ***, P<0.001 (Student's *t*-test). (C) The absolute numbers of CXCR5^high^PD-1^high^ cells in spleens, mesenteric LN, and livers from normal and infected mice were calculated. Data are expressed as the mean ± SD of 12 mice from three independent experiments, ***, P<0.001 (Student's *t*-test). (D) Spleens, mesenteric LN, and livers from normal and infected mice were harvested, and CD4^+^ T cells were MACS purified, surface stained with CXCR5-APC, PD-1-PE, ICOS-pecy5.5 antibodies and subsequently intracellular stained with BCL-6 Alexa Fluor 488 antibody. The expression of Bcl6 and ICOS in CXCR5^high^PD-1^high^CD4^+^ T cells and CXCR5^low^PD-1^low^CD4^+^ T cells in spleen, mesenteric LN, and livers was evaluated.

To assess whether Tfh cells are expanded in mice infected with *S. japonicum*, we analyzed the percentages of CXCR5^high^PD-1^high^ CD4^+^ T cells (Tfh cells) [32] in the spleens, lymph nodes, and livers of mice. Result in Figure 1 showed that the percentages and absolute numbers of CXCR5^high^PD-1^high^ CD4^+^ Tfh cells were significantly increased in the spleen, lymph nodes, and liver of *S. japonicum* infected mice (Figure S1, Figures 1A, 1B, and 1C). Tfh cells are also characterized by altered expression of other markers, such as the transcription factor Bcl6 and the costimulatory receptor ICOS [10]. Thus, to further confirm the above CXCR5^high^PD-1^high^ CD4^+^ T cells are Tfh cells, their expression of Bcl6 and ICOS was examined. Result in Figure 1D showed that CXCR5^high^PD-1^high^ CD4^+^ Tfh cells expressed high levels of Bcl6 and ICOS compared to non-Tfh cells in the spleen, lymph nodes, and liver of *S. japonicum* infected mice.

### Tfh cells promote the granuloma formation in mice infected with *S. japonicum*

**Figure 2:**
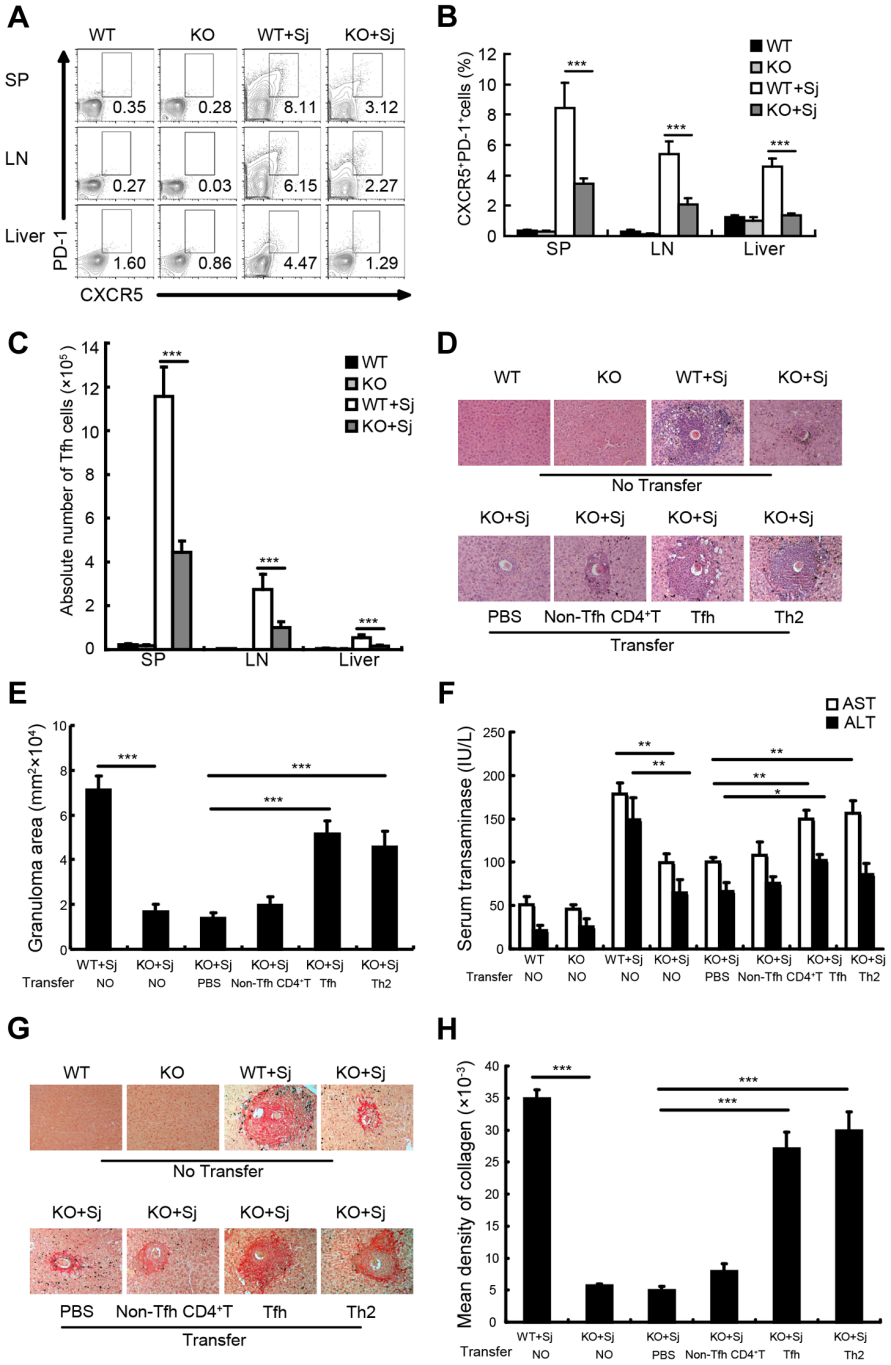
Tfh cells promote liver pathology. (A) Mouse spleens, mesenteric LN, and livers from WT and ICOSL KO mice infected with or without *S. japonicum* were harvested, and cells were stained with CD3-percpcy5.5, CD4-FITC, CXCR5-APC, and PD-1-PE antibodies. Data shown are gated on CD3^+^CD4^+^ cells. Numbers represent the frequency of the boxed population within the CD4^+^ T cell population; (B) Data are expressed as the mean ± SD of 18 mice from three independent experiments, ***, P<0.001 (Student's *t*-test); (C) The absolute numbers of CXCR5^high^PD-1^high^ cells in spleens, mesenteric LN, and livers from WT and ICOSL KO mice infected with or without *S. japonicum* were calculated. Data are expressed as the mean ± SD of 18 mice from three independent experiments, ***, P<0.001 (Student's *t*-test); (D) Three weeks after adoptive transfer of none, PBS, non-Tfh CD4^+^ T cells, Tfh cells, or Th2 as described in Materials and Methods, liver sections from *S. japonicum*-infected ICOSL KO recipient mice were Paraffin-embedded, formalin fixed and stained with H&E. Images shown are representative of two independent experiments. Original magnification, ×100; (E) For each mouse, the sizes of 30 granulomas around single eggs were quantified with AxioVision Rel 4.7. Data are expressed in area units. Values are given as mean ± SD of 12 mice from two independent experiments, ***, P<0.001 (Student's *t*-test), compared with control mice; (F) Serum samples were collected from mice three weeks after adoptive transfer of none, PBS, Tfh cells, non-Tfh CD4^+^ T cells, or Th2 cells. Levels of serum ALT/AST were determined. Data are expressed as the mean ± SD of 12 mice from two independent experiments, **, P<0.01; *, P<0.05 (Student's *t*-test); (G) Paraffin-embedded sections were stained with sirius red. Images shown are representative of two independent experiments. Original magnification, ×100; (H) The mean optical density of collagen fibers by sirius red staining was digitized and analyzed on Image-Pro Plus software. Values are given as mean ± SD of 12 mice from two independent experiments, ***, P<0.001 (Student's *t*-test), compared with control mice.

The interaction between ICOS and ICOSL is required for Tfh cell differentiation, but not for Th2 cell differentiation and migration into nonlymphoid tissues, and ICOSL knockout (KO) mice were widely used as a Tfh cell deficiency model [17], [33]–[35]. To investigate whether Tfh cells are required for liver granuloma formation, we infected ICOSL KO mice that lack Tfh cells [8] with *S. japonicum*. Results showed that the percentages (Figures 2A and 2B) and absolute numbers (Figure 2C) of CXCR5^high^PD-1^high^ Tfh cells were significantly reduced in the spleen, lymph nodes, and liver of ICOSL KO mice compared to wild-type (WT) mice. Meanwhile, the area of granuloma and the severity of the fibrosis in livers of Tfh cell-deficient mice were significantly reduced than that in WT mice (Figures 2D, 2E, 2G and 2H) and liver injury was milder in ICOSL KO mice (Figure 2F). Interestingly, our results showed that schistosome antigen specific IgG and IgG1 production was greatly reduced in ICOSL KO infected mice (Figure S2), which consistent with the published data that proved the critical role of Tfh cells in production of antibody.

To investigate the impact of ICOSL deficiency on other CD4^+^ T cell subsets involved in granuloma formation, we detected the percentages of Th1, Th2, Th17 and Treg cells in spleen, lymph nodes and liver in mice infected with *S. japonicum*. We found that both Th2 and Th17 cells, which were reported to upregulate hepatic granuloma formation [4], [5], showed no significant difference between ICOS KO mice and their WT controls with or without infection (Figures S3A and S3B). Although the significant differences of Th1 and Treg cells in spleen and/or lymph nodes between ICOS KO mice and their WT controls were observed before infection, both Th1 and Treg cells, which were reported to downregulate hepatic granuloma formation [5], [7], were less in infected ICOSL KO mice livers (Figures S3C and S3D). These data suggest that significantly reduced Tfh cells may account for the amelioration of liver pathology in ICOSL KO mice infected with *S. japonicum.*

To further elucidate the contribution of Tfh cells to liver granuloma formation in *S. japonicum*-infected ICOSL KO mice, Tfh cells, non-Tfh, or Th2 control cells were purified from *S. japonicum*-infected WT mice and adoptively transferred into ICOSL KO mice 5 weeks after *S. japonicum* infection. Result in Figure S4 showed that eGFP^+^ Tfh cells still expressed the molecular markers of CXCR5 and PD-1 three weeks post-transfer. Results showed that compared with phosphate buffered saline (PBS) group, granuloma size and the levels of serum ALT/AST were not statistically significantly increased in mice receiving non-Tfh cells (comprised of pooled antigen-specific Th1/Th2/Th17/Treg cells), which suggests that pooled antigen-specific CD4^+^ T cells may not be sufficient to promote the granuloma formation and liver injury. Of note, the area of granuloma and severity of fibrosis were significantly exacerbated after adoptive transfer of Tfh or Th2 cells into infected KO mice (Figures 2D, 2E, 2G and 2H). Moreover, the adoptive transfer of Tfh or Th2 cells resulted in a significant increase in the levels of serum ALT/AST (Figure 2F), compared with control mice injected with PBS alone or non-Tfh control cells. These results suggest that Tfh cells play a pivotal role in promotion of the liver granuloma formation and liver injury, although we did not directly rule out the possibility that it might be partially resulted from more antigen-specific CD4^+^ T cells in “Tfh group” than that in the “non-Tfh control group”.

Taken together, these data suggest that Tfh cells contribute to liver pathology in mice infected with *S. japonicum*.

### Tfh cells are recruited to the liver in *S. japonicum*-infected mice

**Figure 3:**
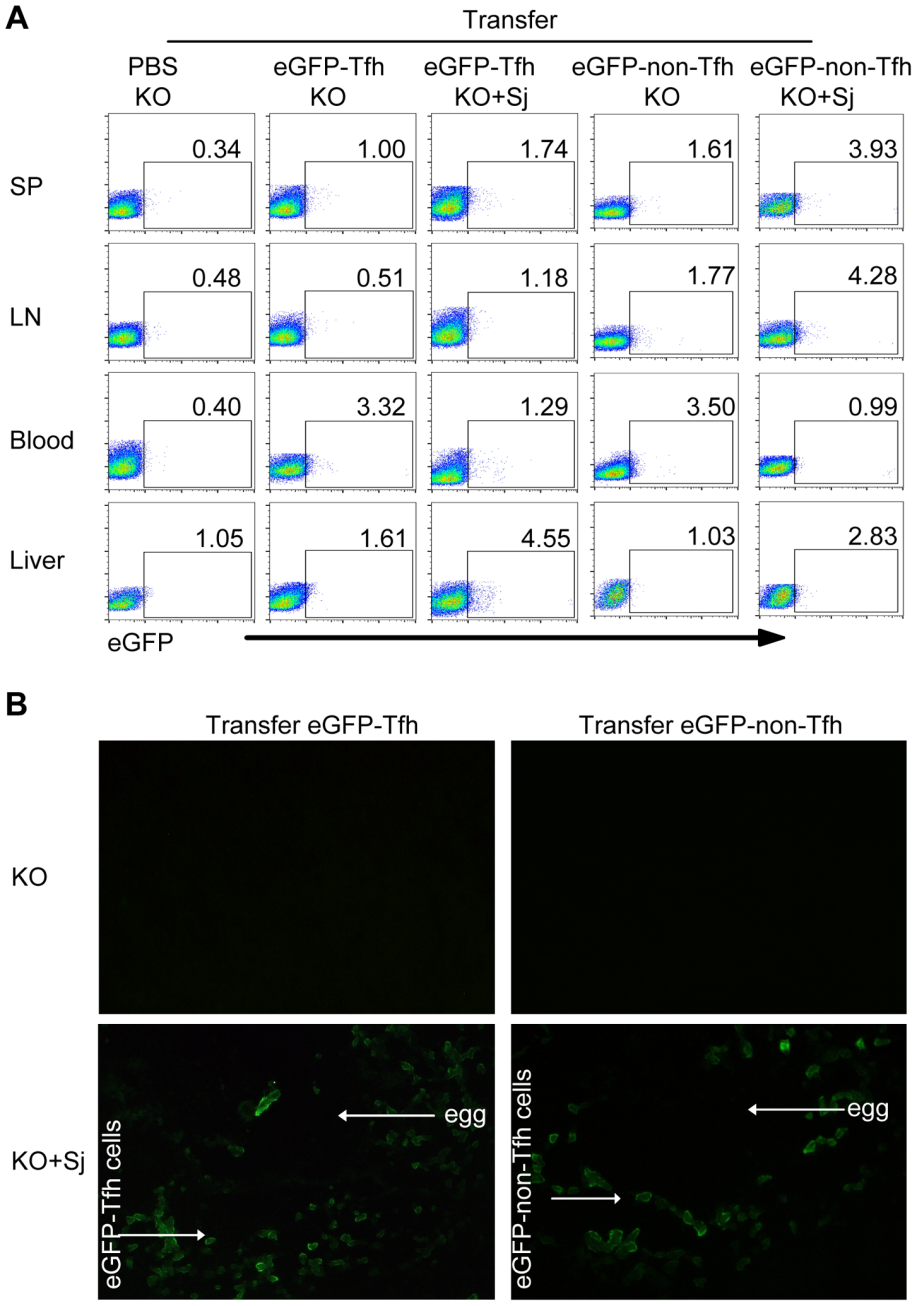
Tfh cells are recruited to the liver in infected mice. Five weeks after *S. japonicum* infection, ICOSL KO mice were received the PBS, eGFP^+^ Tfh cells or eGFP^+^ non-Tfh cells as described in Materials and Methods. (A) Flow cytometric pseudocolor plots of eGFP^+^ cells from normal and infected mice recipients 3 days after adoptive transfer of the PBS, eGFP^+^ Tfh or eGFP^+^ non-Tfh cells. The numbers in the plots represent percentages; (B) Representative of liver slices from normal and infected mice recipients 3 days after adoptive transfer. Original magnification, ×400.

To determine whether Tfh cells can be recruited to the liver of infected mice, we adoptively transferred eGFP^+^ Tfh cells or eGFP^+^CD44^+^CXC5^−^PD-1^−^CD4^+^ T (eGFP^+^ non-Tfh) cells from eGFP mice into *S. japonicum*-infected ICOSL KO mice. Three days after transfer, by flow cytometry and fluorescence microscopy, we found that most eGFP^+^ Tfh cells were observed in the blood circulation in uninfected ICOSL KO mice. However, in *S. japonicum*-infected ICOSL KO mice, most eGFP^+^ Tfh cells appeared in the liver (Figures 3A and 3B). The eGFP^+^ Tfh cells were limited to appear in the granulomas forming around newly deposited eggs within 3 days after transfer. Interesting, most eGFP^+^ non-Tfh cells, which include Th1/Th2/Th17/Tregs, also appeared mainly in the blood circulation in uninfected mice, while eGFP^+^ non-Tfh cells were mainly in spleen, lymph nodes and liver in *S. japonicum*-infected mice. In addition, more GFP^+^ Tfh cells were found in *S. japonicum*-infected mice liver than eGFP^+^ non-Tfh cells (Figures 3A and 3B). These data suggest that Tfh cells, which may be induced by APCs probably including B cells and/or DCs in lymph nodes and spleen as previously reported, are recruited to the mice liver and promote the granuloma formation around eggs after infected with *S. japonicum*.

### Macrophages from *S. japonicum*-infected mice drive Tfh-cell generation

**Figure 4:**
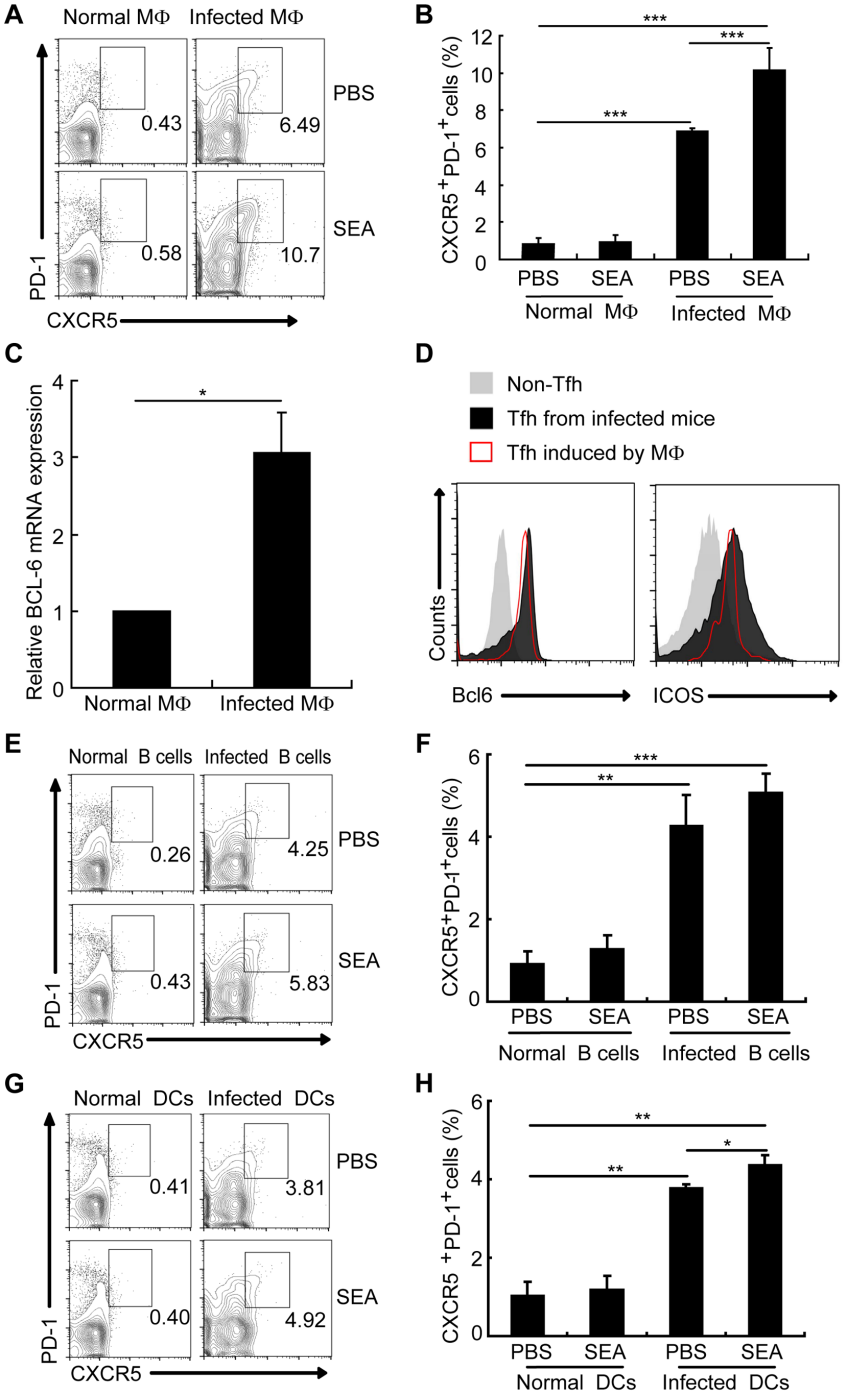
Macrophages drive Tfh cell generation. Expression of CXCR5 versus PD-1 on CD4^+^ T cells (gated as CD3^+^CD4^+^) after co-culture of normal mice derived CD4^+^ T cells with macrophages (A, B), B cells (E, F), and DCs (G, H) from normal or infected mice in the presence or absence of SEA. Numbers represent the frequency of the boxed population within the CD4^+^ T cell population; (C) Quantitative RT-PCR analysis of the expression of *BCL-6* mRNA in CD4^+^ T cells cultured with or without macrophage from infected mice. ***, P<0.001; **, P<0.01; *, P<0.05 (Student's *t*-test); (D) The expression of Bcl6 and ICOS was evaluated as previous described in CXCR5^low^PD-1^low^CD4^+^ T cells and CXCR5^high^PD-1^high^CD4^+^ T cells directly isolated from *S. japonicum*-infected mice or *in vitro* induced by macrophages from *S. japonicum*-infected.

Macrophages make up approximately 30% of total liver granuloma cells in *S. japonicum*-infected mice, and most of these macrophages (50–90%) display Ia antigens, acting as professional APCs [36]. Considering macrophages co-locate in spleen and lymph nodes with other two important APCs B cells and DC, and play an important role as APC in induction of the differentiation of Th1 [37], Th2 [38], Th17 [39], [40], and Treg cells [41]–[43], it was interesting to investigate whether macrophages had an effect on the generation of Tfh cells. To address this question, we cultured CD4^+^ T cells together with macrophages from normal or infected mice in the presence or the absence of soluble egg antigens (SEA) extracted from *S. japonicum* eggs. SEA is a mixture of antigens including numerous molecules of protein, glycoprotein, glycolipid, lipoprotein and saccharide. SEA provides polyclonal stimulations to immune cells including APC and CD4^+^ T cells. Results showed that CD4^+^ T cells not only increased the surface expression of CXCR5 and PD-1 (Figures 4A and 4B) but also upregulated transcripts of the Tfh cell “master regulator” *Bcl-6* and ICOS (Figures 4C and 4D) when exposed to macrophages from the infected mice.

B cells and DCs, the other two important professional APCs co-located in spleen and lymph nodes with macrophages, are reported to have the ability to induce Tfh-cell development. Result showed that compared with B cells (Figures 4E and 4F) and DCs (Figures 4G and 4H) from infected mice, macrophages from infected mice induced a higher frequency of CXCR^high^PD-1^high^CD4^+^ T cells. These results prove again that macrophages from *S. japonicum*-infected mice have the similar ability as B cells and DCs to drive the generation of CD4^+^ T cells into Tfh cells.

### Macrophages drive Tfh-cell generation through a cell-cell contact-dependent mechanism

**Figure 5:**
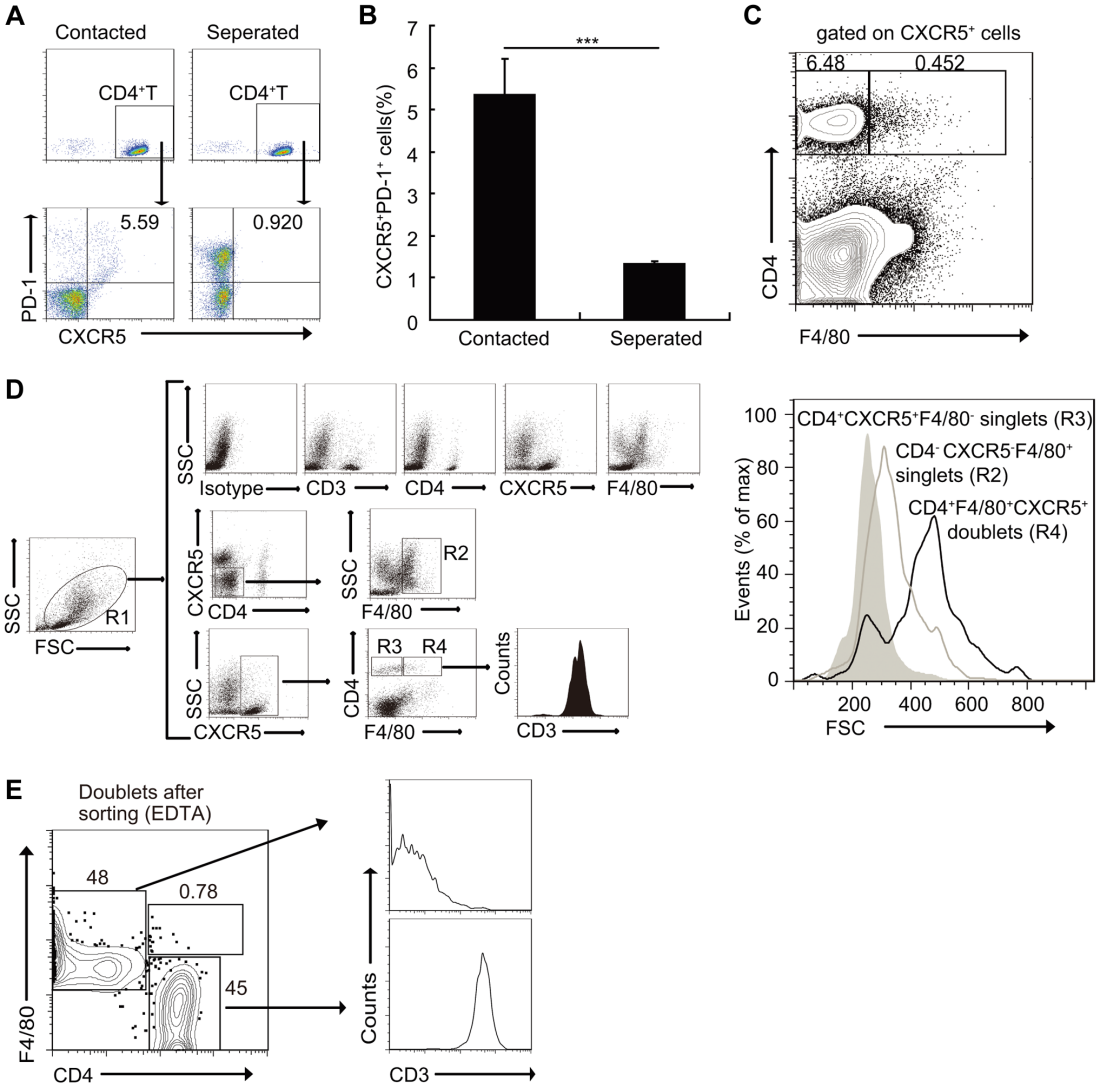
Macrophages drive Tfh cell development through a cell-cell contact-dependent mechanism. (A, B) In a transwell system, macrophages from infected mice were cultured in triplicate wells in the lower chambers. CD4^+^ T cells from normal mice were cultured in the upper chambers. Expression of CXCR5 versus PD-1 on CD4^+^ T cells (gated as CD3^+^CD4^+^) after co-culture. Data are representative of two independent experiments. Numbers represent the frequency of the boxed population within the CD4^+^ T cell population. ***, P<0.001 (Student's *t*-test); (C) Flow cytometry of CD4^+^CXCR5^+^F4/80^+^ doublets in the spleens of *S. japonicum*-infected mice. Data are representative of three experiments with three mice in each group; (D) Gating strategy defining CD4^+^CXCR5^+^F4/80^−^ singlet cells (R3), CD4^−^CXCR5^−^F4/80^+^ singlet cells (R2) or CD4^+^CXCR5^+^F4/80^+^ doublets (R4), (*D right*). Size (forward scatter (FSC)) of CD4^+^CXCR5^+^F4/80^−^ singlet cells, CD4^−^CXCR5^−^F4/80^+^ singlet cells or CD4^+^CXCR5^+^F4/80^+^ doublets in the spleens of *S. japonicum*-infected mice (*D left*). Data are representative of three experiments. (E) Flow cytometry of CD3^+^CD4^+^CXCR5^+^F4/80^+^ doublets sorted from the pooled spleens from three *S. japonicum*-infected mice and then treated with 2 mM EDTA. Data are representative of three independent experiments.

In parallel cultures, CD4^+^ T cells were separated from macrophages by a porous (0.4-µm) membrane in otherwise identical conditions. Results showed that compared to the co-culture group, the separation of CD4^+^ T cells from macrophages increased only the expression of PD-1, instead of CXCR5 (Figures 5A and 5B). Moreover, a small fraction of CXCR5^+^CD4^+^ T cells sorted from *S. japonicum*-infected mice spleens also expressed the macrophage marker F4/80, which suggested that these may represent stable macrophage-T cell conjugates (Figure 5C). In addition, the mean FSC value of the cells which expressed both T cell and macrophage markers was approximately 500, compared to T cells (CD4^+^CXCR5^+^F4/80^−^) and macrophages (CD4^−^CXCR5^−^F4/80^+^) that were approximately 200 and 300, respectively (Figure 5D), which suggested again that they were macrophage-T cell conjugates. Data showed that almost all CXCR5^+^CD4^+^F4/80^+^ cells expressed the T cell antigen receptor marker CD3, and further suggested that they were macrophage-T cell conjugates (Figure 5D). Next, we sorted the CD3^+^CXCR5^+^CD4^+^F4/80^+^ cells to high purity and then treated the cells with EDTA to dissociate cell-cell contacts. The resulting single cells segregated into approximately equal numbers of CD4^+^ and F4/80^+^ single-positive populations (Figure 5E), which further confirmed the stable conjugates. The macrophage-T cell conjugates in *S. japonicum*-infected mice livers were also detected (Figure S5). Taken together, these data suggest that direct cell-cell contact is required for Tfh cell development driven by macrophages, and also suggest a physiological role of these conjugates *in vivo*.

### Macrophages drive Tfh-cell generation dependent on ICOSL signaling

**Figure 6:**
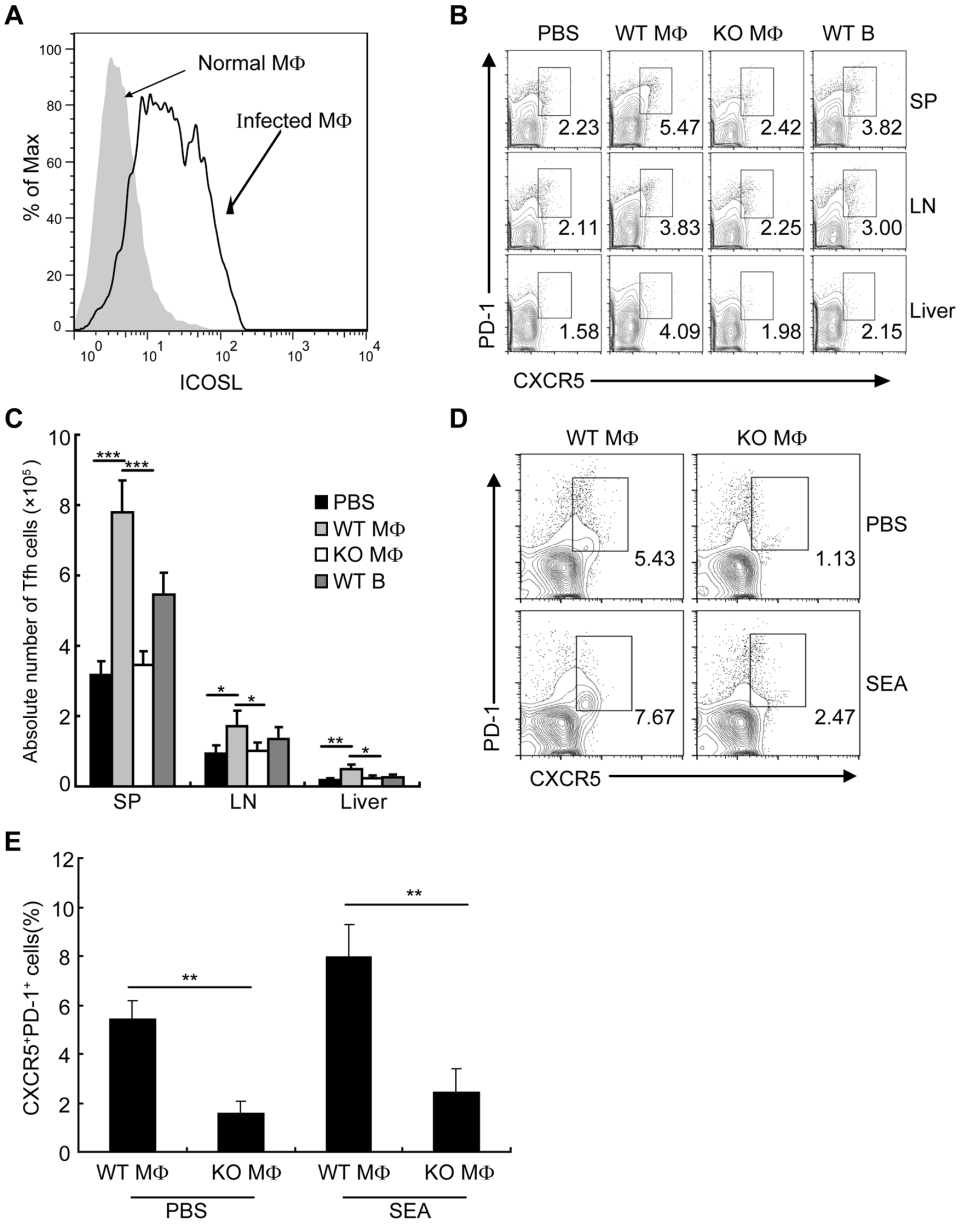
Macrophages drive Tfh-cell development dependent on ICOSL signaling. (A) Flow cytometric histogram of ICOSL^+^ PM cells (gated on F4/80^+^ cells) from normal and infected mice. Data are representative of three experiments with 3 mice in each group; (B) Seven days after adoptive transfer of the WT B, WT macrophage or ICOSL^−/−^ macrophage cells from infected mice, spleens, mesenteric LN, and livers cells from infected ICOSL KO recipient mice were harvested and stained as previously described for CXCR5 and PD-1 expression. Data shown are gated on CD3^+^CD4^+^ cells. Numbers represent the frequency of the boxed population within the CD4^+^ T cell population. Data are representative of two experiments with three mice in each group; (C) Absolute numbers of CXCR5^+^PD-1^+^ Tfh cells in spleens, mesenteric LN, and livers of the infected ICOSL KO recipient mice seven days after adoptive transfer of the WT B, WT macrophage or ICOSL^−/−^ macrophage cells are shown. Data are representative of two experiments with three mice in each group. ***, P<0.001; **, P<0.01; *, P<0.05 (Student's *t*-test); (D) Expression of CXCR5 versus PD-1 on CD4^+^ T cells (gated as CD3^+^CD4^+^) after co-culture of CD4^+^ T cells from normal mice with WT or ICOSL^−/−^ macrophages from infected mice in the presence or absence of SEA. Numbers represent the frequency of the boxed population within the CD4^+^ T cell population. Data are representative of three experiments with 3 mice in each group; (E) Data are expressed as the mean ± SD of three experiments with 3 mice in each group. **, P<0.01 (Student's *t*-test).

We found that the expression of ICOSL on DCs from *S. japonicum*-infected mice was significantly increased (Fig. S6), which supported by the report that the expression of ICOSL on DCs is required for the first step of Tfh-cell induction, and is further required for T cell–B cell interactions for the maintenance of Bcl-6 expression [33]. However, it is unknown whether ICOSL is required for Tfh cell generation driven by macrophages. To address this issue, we detected the expression of ICOSL by macrophages and found that macrophages from *S. japonicum*-infected mice expressed higher amounts of ICOSL than macrophages from normal mice (Figure 6A). We adoptively transferred WT macrophages, ICOSL KO macrophages, or WT B cells as control from *S. japonicum*-infected mice into *S. japonicum*-infected ICOSL KO mice. Consistent with published data [44]–[46], our result showed that macrophages were still alive in recipient mice one week after transferring (Figure S7A). Furthermore, our study demonstrated that some of the adoptively transferred-macrophages migrated into spleen, lymph nodes and liver in *S. japonicum*-infected recipient mice one week post-transfer (Figure S7B). Seven days after transfer, we detected the higher percentage and absolute number of Tfh cells in the spleens, lymph nodes, and livers of mice receiving WT macrophages compared to mice receiving ICOSL KO macrophages (Figures 6B and 6C), indicating that macrophages from infected mice sufficiently promote the Tfh cell formation *in vivo*. Finally, we cultured normal mice derived CD4^+^ T cells together with macrophages from *S. japonicum*-infected WT or ICOSL KO mice, with or without antigen. The results showed that consistent with the result in Figure 4, CD4^+^ T cells increased the surface expression of CXCR5 and PD-1 when exposed to WT macrophages but not ICOSL KO macrophages (Figures 6D and 6E). Thus, these data indicate that ICOSL expressed by macrophages is essential for Tfh cell induction driven by macrophages.

### CD40L regulates ICOSL expression on macrophages for Tfh cell generation

**Figure 7:**
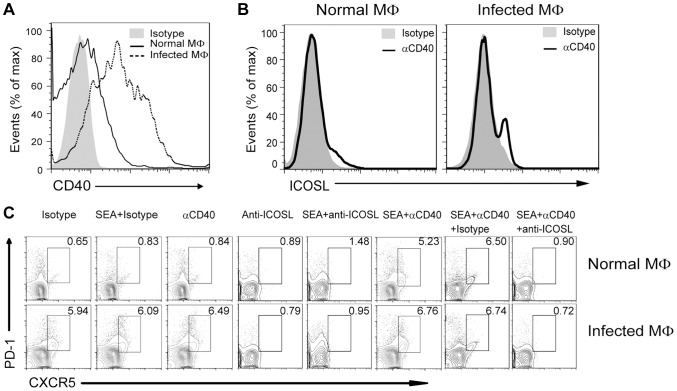
CD40L regulates ICOSL expression in macrophages for Tfh-cell development. (A) Freshly isolated PM of normal and infected mice were stained with F4/80-FITC and CD40-PE and analyzed by flow cytometry. Data are representative of three experiments with 3 mice in each group; (B) PM from normal or infected mice were cultured *in vitro* for 72 h either in the presence of an agonistic anti-CD40 antibody or its isotype control antibody. The intensity of ICOSL surface expression was measured by flow cytometry after F4/80-FITC and ICOSL-PE staining; (C) Expression of CXCR5 versus PD-1 on CD4^+^ T cells (gated as CD3^+^CD4^+^) after co-culture of normal mice-derived CD4^+^ T cells with normal or *S. japonicum*-infected mice-derived macrophages for 3 days with or without SEA in the presence or absence of agonistic anti-CD40 antibody and anti-ICOSL antibody or isotype-matched control antibody. Numbers represent the frequency of the boxed population within the CD4^+^ T cell population. Data are representative of three experiments with 3 mice in each group.

In addition to the ICOSL expressed on DCs, CD40L–CD40 interactions between CD4^+^ T cells and B cells or DCs is also required for Tfh cell generation [26]. Considering the level of ICOSL on B cells is regulated by the noncanonical NF-κB pathway, which can be triggered by signals from a subset of tumor necrosis factor receptor (TNFR) family members, including B-cell-activating factor and CD40 [47], we hypothesized that CD40/CD40L signaling regulates Tfh cell generation by regulation of ICOSL expression on macrophages. To this end, we examined the expression of CD40 by macrophages and found that macrophages from infected mice had a higher level of CD40 than macrophages from normal mice (Figure 7A), Incubation of macrophages with agonist anti-CD40 antibody led to the potent induction of ICOSL expression *in vitro* (Figure 7B). In addition, agonist anti-CD40 antibody treatment had increased expression of surface CXCR5 and PD-1 on CD4^+^ T cells when exposed to macrophages from normal or infected mice in the presence of SEA. However, after treatment with anti-ICOSL antibody to block ICOS-ICOSL signaling in co-culture system, macrophages could not induce the expression of surface CXCR5 and PD-1 on CD4^+^ T cells (Figure 7C). Taken together, the data in figures 6 and 7 suggest that CD40L–CD40 signaling regulates ICOSL expression on macrophages for Tfh-cell generation.

## Discussion

The appropriate granulomatous lesions formation favors the survival of both host and parasite after *S. japonicum* infection. Thus, rational treatment of patients with schistosomiasis requires a better understanding of the mechanism of granuloma formation to prevent excessive granulomatous lesions. In this study, we reported a novel role for Tfh cells in the development of liver pathology in *S. japonicum*-infected mice and uncovered an unappreciated function of macrophages in Tfh induction.

Pathology induced by *S. japonicum* infection in host tissues, especially in the liver, is predominantly caused by the immune responses in response to schistosome eggs. Through an unclear mechanism, CD4^+^ T cell response induced by egg antigens orchestrates the development of granulomatous lesions around individual eggs trapped in host liver, which are composed of macrophages, eosinophils, and CD4^+^ T cells [3]. In this study, we found that the percentages and absolute numbers of Tfh cells in the spleen, lymph nodes, and liver were significantly increased 8 weeks after *S. japonicum* infection. However, whether Tfh cells, as the other CD4^+^ T cell populations (Th1, Th2, Th17 and Treg cells) do, contribute to the liver pathology in mice with schistosomiasis remains to be explored.

Our result showed that Tfh cells had a profound effect on the formation of liver pathology in *S. japonicum*-infected mice. Using ICOSL KO mice, in which the generation of Tfh cells has been proven to be defective, we demonstrated for the first time that Tfh cells participate in the formation of hepatic granuloma in mice infected with *S. japonicum*, although we still can not fully rule out the possibility that ICOSL also plays a possible role in the generation of the other effector cells and impacts the formation of hepatic granuloma. In addition, we observed significantly lower levels of parasite antigen specific IgG and IgG1 antibodies in *S. japonicum* infected ICOSL KO mice. It is widely accepted that antibodies produced by B cells are dispensable for granuloma formation in mice infected with *S. mansoni* or *S. japonicum* [48]–[50]. Thus, the typical function of Tfh cells with regard to providing help to B cells to produce antibodies may not be involved in granuloma formation. Of note, IL-4-producing CD4^+^ T cells are generally considered to be essential for granuloma formation during schistosome infection [3], while IL-21 could promote Th2 responses and upregulate granuloma inflammation [51], [52]. Previous reports in mice showed that Tfh cells were significantly induced during *S. mansoni* infection [19] and that most IL-4-producing CD4^+^ T cells in reactive lymph nodes produced in response to *S. mansoni* antigens are Tfh cells [20]. Studies also reported that Tfh cells produce IL-21 [9], [53]. These data indicate a potential role and mechanism for Tfh cells in liver granuloma formation in schistosome infection via the production of IL-4 and IL-21. In addition, our recent unpublished data suggests that production of CXCL12 by Tfh cells may also involve in the upregulation of the granuloma formation. We have found that most Tfh cells during *S. japonicum* infection produced a high level of CXCL12 (unpublished data), which is the ligand for CXCR4 on eosinophils, the most predominant cell types within granulomas, and is required for eosinophil migration [54]. Consistent with this notion, eosinophils were dramatically decreased in the liver of ICOSL KO mice (unpublished data), suggesting that Tfh cells may also regulate granuloma formation by recruiting eosinophils to the liver in mice infected with *S. japonicum*. However, the exact mechanism for the involvement of Tfh cells in the formation of granuloma during *S. japonicum* infection requires further clarification.

It is widely accepted that the generation of Tfh cells from naïve precursors typically involves interactions with antigen-presenting cells such as DCs within lymphoid tissues including spleen and the draining lymph nodes [32]. In addition, studies demonstrate that Tfh cells, which express CXCR5, can migrate in response to CXCL13 and relocate to the follicles of lymphoid tissues. Thus, a high percentage of Tfh cells have been detected in the spleen and lymph nodes in various mice models [8], [17], [55], [56]. However, Tfh cells have also been detected in nonlymphoid tissues, such as peripheral blood, in patients with immune-active chronic hepatitis B or systemic lupus erythematosus [57]–[59], in the liver of hepatitis C virus (HCV)-infected patients [60] and mouse models [61]. In addition to in the spleen and lymph node, Tfh cells were also found in the liver from *S. japonicum*-infected mice in our study. Although our results obtained from Tfh cells transfer experiment proved that Tfh cells in peripheral were recruited to the liver and promoted the formation of liver granulomas, the mechanism of their cellular origin and migration still needs further illustration.

Macrophages play an important role in host immune responses against *S. japonicum* infection. In addition to their role as phagocytes, most of these macrophages (50–90%) display Ia antigens, and express MHC and costimulatory molecules, acting as professional APCs by processing and presenting SEA to CD4^+^ T cells [36]. A large number of macrophages, DCs and B cells co-locate in the marginal zone of spleen or lymph nodes, and it is well known that after activation, the entry of APCs to the white pulp, in particular to the T-cell zone, is an important step in the initiation of CD4^+^ T cell response [62]. Although the roles of B cells and DCs in the generation of Tfh cells [21], [26], and the role of macrophages in the induction of Th1 [37], Th2 [38], Th17 [39], [40], and Treg cells [41]–[43] have been documented in previous reports, no data have been published to address whether macrophages are also involved in the generation of Tfh cells. In addition to the reported ability of macrophages to induce generation of Th1, Th2, Th17 and Treg cells [63], [64], our observations show that macrophages also have ability to aid the generation of Tfh cells during *S. japonicum* infection, which is at least partially supported by our *in vitro* co-culture experiments and the macrophages transfer experiment. However, our *in vitro* experiment can not rule out the proliferation of the Tfh cells stimulated by macrophages also contribute to the increase of the Tfh cells. Although increasing evidence supports the concept that the liver is a secondary lymphoid organ, acting as a site of T cell activation and differentiation [65], whether Tfh cells can be induced by macrophages in liver is worthy of further research.

Our results suggest that CD4^+^ T cell-macrophage conjugates are necessary for Tfh cell generation, and establish a physiological relevance for these conjugates *in vivo*. Thus, our data are consistent with a model in which B cell–T cell conjugates regulate Tfh-cell generation [17], although the exact mechanism of T cell-macrophage conjugates remains to be explored. Unexpectedly, the separation of CD4^+^ T cells from macrophages considerably increased the expression of PD-1, which has commonly been used as a marker for T cell exhaustion in both mouse and human infections [66]–[68], suggesting that macrophage-CD4^+^ T cell conjugates may contribute to prevent the exhaustion of CD4^+^ T cells. However, the characterization of the potential CD4^+^ T cell exhaustion needs to be investigated.

Up to now, the mechanisms of the induction of CXCR5 and PD-1 in CD4 T cells are still unclear. Previous studies have shown that ICOS–ICOSL engagement between B cells and CD4^+^ T cells activates PI3 kinase (PI3K) signaling and provides signals to CD4^+^ T cells for the initiation and maintenance of Tfh differentiation and expression of CXCR5 and PD-1 [33], [56], [69]. Our data consistently showed that Tfh cell generation driven by macrophages is also dependent on ICOSL signaling. Intriguingly, our results showed that the level of ICOSL expression on macrophages from normal mice was much lower than that from infected mice, providing a possible explanation as to why macrophages from infected mice, rather than those from normal mice, significantly induced Tfh cell generation both *in vitro* and in adoptive transfer experiment. Thus, macrophages may represent a novel subset of APCs that prime Tfh generation in mice infected with *S. japonicum*.

CD40L signaling in CD4^+^ T cells is critical for T cell priming and maintenance in most *in vivo* contexts [70], [71]. CD40L–CD40 interactions, with either B cells or DCs, are required for Tfh cell development [26]. However, the exact mechanism of CD40–CD40L signaling in the generation of Tfh cells has remained unclear. For the first time, our results suggest that the activation of CD40–CD40L signaling upregulates ICOSL expression on macrophages, and subsequently facilitates Tfh cell generation.

In summary, except for Th1, Th2, Th17, and Treg cells, we have demonstrated a novel role of Tfh cells in the granulomatous pathology in mice infected with *S. japonicum*, which challenges the existing paradigm that Tfh cells are specialized for providing B cell help. We have also shown that macrophages are able to promote the generation of Tfh cells in a cell-cell contact manner and regulated by CD40–CD40L signaling. In addition, our data indicate a therapeutic potential to target a Tfh cell induction or migration axis in liver pathology caused by *S. japonicum* infection.

## Materials and Methods

### Ethics statement

Animal experiments were performed in strict accordance with the Regulations for the Administration of Affairs Concerning Experimental Animals (1988.11.1), and all efforts were made to minimize suffering. All animal procedures were approved by the Institutional Animal Care and Use Committee (IACUC) of Nanjing Medical University for the use of laboratory animals (Permit Number: NJMU 09-0163).

### Mice, infection and antigen preparation

Eight-week-old male C57BL/6J mice and eGFP C57BL/6J mice were purchased from the SLAC Laboratory (Shanghai, China) and Model Animal Research Center of Nanjing University (Nanjing, China), respectively. Eight-week-old male ICOSL^−/−^ C57BL/6J mice were obtained from Soochow University (Suzhou, China). Animals were kept under specific pathogen-free conditions and were used at 8–12 weeks of age. In the infection experiments, mice were infected percutaneously with 12 *S. japonicum* cercariae (Chinese mainland strain) obtained from infected *Oncomelania hupensis* snails purchased from the Jiangsu Institute of Parasitic Diseases (Wuxi, China). SEA was obtained from purified and homogenized *S. japonicum* eggs. The protein concentration of SEA was determined using a bicinchoninic acid (BCA) Protein Assay kit (Bio-rad, Richmond, CA).

### Flow cytometry

Macrophage-T cell doublets were sorted with a FACSAria cell sorter (BD Biosciences). Conjugates were dissociated with 2 mM EDTA and vigorous vortexing. Single cell suspensions were prepared by teasing spleens, inguinal and mesenteric lymph nodes (LN) and blood in PBS containing 1% EDTA followed by red blood cell (RBC) lysis. Hepatic lymphocytes were prepared as described previously with some modifications [72]–[75]. In brief, for preparation of single cell suspensions of hepatic lymphocytes, infected or control mice livers were perfused via the portal vein with PBS. The excised liver was cut into small pieces and incubated in 10 ml of digestion buffer (collagenase IV/dispase mix, Invitrogen Life Technologies, Carlsbad, CA) for 30 min at 37°C. The digested liver tissue was then homogenized using a Medimachine with 50-µm Medicons (Becton Dickinson, San Jose, CA) according to the manufacturer's instructions. The liver suspension was resuspended in 5 ml PBS and then placed on a lympholyte M (Cedarlane, Ontario, Canada) overlay in a 1∶1 ratio. Cells were spun at 2,200 rpm for 20 minutes, collected from PBS/Lympholyte M interface, washed and suspended in PBS containing 1% EDTA. For surface staining, 2×10^6^ cells per 100 µl were incubated for 30 min at 4°C with the following fluorescently labeled monoclonal antibodies: CD3e-Percp-cy5.5 (eBioscience, San Diego, CA), CD3e-PE (eBioscience), CD4-FITC (eBioscience), CD4-PE-Cy7 (eBioscience), CXCR5-APC (BD Pharmingen, San Diego, CA), PD-1-PE (eBioscience), ICOS-PE-Cy5 (eBioscience), F4/80-FITC (eBioscience), F4/80-PE (eBioscience), CD40-PE (eBioscience), CD44-Percp-cy5.5 (eBioscience), ST2-APC (eBioscience), and ICOSL-PE (eBioscience). After staining of surface markers, the cells were permeabilized with cold Fix/Perm Buffer, and Fc receptors of cells were blocked with anti-mouse CD16/32 (eBioscience) for 15 min. The Bcl6-Alexa Fluor 488 (BD Pharmingen) was then added and incubated for 30 min at 4°C. The cells were then washed twice in wash buffer before analysis. The details of measurement of Th1, Th2, Th17, and Treg cells by flow cytometry are provided in Text S1.

### Macrophage isolation and culture

Peritoneal macrophages (PM) were prepared as described previously with some modifications [76]. Briefly, mice were sacrificed by cervical dislocation, and 7 ml of ice-cold PBS containing 1% fatal bovine serum (FBS) and 50 µg/ml penicillin-streptomycin (Sigma-Aldrich, St. Louis, MO) was injected into the abdominal cavity. The medium containing peritoneal exudates cells (PEC) was recollected and transferred to sterile plastic tubes. The suspended cells were centrifuged at 1500 rpm for 5 min at 4°C, and the cells were resuspended in PBS with 1% FBS. The cells (2×10^6^ cells/well) were then seeded into 12-well culture plates (Costar, Cambridge, MA) and were allowed to adhere for 2–3 h at 37°C with 5% CO_2_. Non-adherent cells were removed by washing the wells with sterile PBS six times, and the remaining monolayers were all PM. Cell viability was ≥90% in all experiments. PM were either directly subjected to flow cytometry analysis or stimulated *in vitro* by anti-mouse CD40 antibody (500 ng/ml; Biolegend, San Diego, CA) for 3 days.

### *In vitro* CD4^+^ T cell differentiation

CD4^+^ T cells, B cells, or DCs were MACS purified from the spleens of normal mice or infected mice using a CD4^+^ T cell negative-isolation kit (Miltenyi Biotec, Auburn, CA), CD45R (B220) MicroBeads (Miltenyi Biotec), or CD11c MicroBeads (Miltenyi Biotec), respectively. Purified CD4^+^ T cells (2×10^6^ cells/well) from normal mice were incubated in triplicate wells with PM (2×10^5^ cells/well), B cells (2×10^5^cells/well), or DCs (2×10^5^cells/well) from normal or *S. japonicum*-infected mice for 3 days with or without SEA (20 µ/ml) in the presence or absence of agonistic anti-CD40 antibody (500 ng/ml; Biolegend). Anti-ICOSL antibody (5 µ/ml; eBioscience) or isotype-matched control antibody (5 µ/ml; eBioscience) were used to block ICOS-ICOSL signaling in co-culture system. Then, CD4^+^ T cells were collected and examined using surface staining. All experiments were repeated once.

### Adoptive transfer experiments

Spleen and mesenteric LN cells from eGFP C57BL/6J or WT mice 8 weeks after infection with *S. japonicum* were pooled, and CD4^+^ T cells were presorted by using a CD4^+^ T cell negative-isolation kit (Miltenyi Biotec). CD4^+^ T cells were stained with CXCR5-APC and PD-1-PE antibodies, or with CD44-Percp-cy5.5 and ST2-PE antibodies. CXCR5^high^PD-1^high^ Tfh cells, Tfh depleted (non-Tfh control) cells or CD44^+^ST2^+^CD4^+^ T cells (Th2) [77], [78] were FACS purified by using a FACSAria cell sorter (BD Biosciences) to investigate the role of Tfh cells in liver pathology. In addition, eGFP^+^CD4^+^ T cells were stained with CXCR5-APC, PD-1-PE, and CD44-Percp-cy5.5 antibodies. CXCR5^high^PD-1^high^ Tfh cells and CD44^+^CXCR5^low^PD-1^low^ (non-Tfh control) cells were FACS purified by using a FACSAria cell sorter to investigate the recruitment of Tfh cells into liver granuloma. Sorted cells were more than 97% pure. FACS-sorted Tfh, non-Tfh control cells or Th2 cells were resuspended in PBS and injected intraperitoneally (ip) into the ICOSL^−/−^ mice 5 weeks after *S. japonicum* infection (3×10^6^ cells/mouse). A group of mice that did not receive T cells but PBS was used as an additional control (mock transfer). Mice were sacrificed 3 days after transfer to investigate the recruitment of Tfh cells into liver granuloma, or 3 weeks after transfer to investigate the role of Tfh cells in liver pathology, respectively. The details of measurement of Tfh-cell plasticity are provided in Text S1.

PMs were prepared as described above and B cells were sorted using MACS (Miltenyi Biotec). PMs or B cells were resuspended in PBS and injected ip into the mice 8 weeks after *S. japonicum* infection (4×10^6^ cells/mouse). Mice were sacrificed 7 days after transfer to investigate the generation of Tfh cells by macrophages *in vivo*. The details of measurement of survival and migration of macrophages are provided in Text S1.

### Liver pathology

Livers were fixed in 10% neutral buffered formalin. Paraffin-embedded sections (4 µm) were dewaxed and stained with hematoxylin and eosin (H&E) for granulomas analysis or sirius red (Sigma) for fibrosis analysis. For each mouse, the sizes of 30 granulomas around single eggs were quantified using AxioVision Rel 4.7 (Carl Zeiss GmbH, Jena, Germany). Data are expressed in area units. All images were captured at 100× magnification using an Axiovert 200M microscope (Carl Zeiss), and granulomas were analyzed using Axiovision software (Carl Zeiss). Moreover, fibrosis was determined histologically by measuring the intensity of fibrosis in six random (100×) digital images captured from collagen-specific sirius red-stained slides of each mouse using Image-Pro Plus software as previously described. The mean optical density of collagen was determined by dividing integral optical density by the image area [79]. To determine hepatocyte damage, levels of serum alanine transaminase (ALT) and aspartate aminotransferase (AST) were assayed using an Olympus AU2700 Chemical Analyzer (Olympus, Tokyo, Japan).

### Immunohistochemical analysis

Liver was harvested from infected animals and immediately frozen in optimal cutting temperature (OCT) embedding compound over liquid nitrogen. Frozen livers were cut into 8-µm sections on a Leica cryostat and fixed in a mixture of ice-cold 75% acetone/25% ethanol for 5 min. All images were captured using an Axiovert 200M microscope (Carl Zeiss, Inc.) and analyzed using Axiovision software.

### Real-time polymerase chain reaction (RT-PCR)

Total RNA was extracted from cells using an RNeasy Mini Kit. Complementary DNA (cDNA) synthesis was performed using SuperScript RT II and oligo (dT). SYBR Green-based RT-PCR was performed with FastStart Universal SYBR Green Master (Rox) reagents and an ABI PRISM 7300. Relative expression was calculated using the 2^−ΔΔCt^ method normalized to hypoxanthine-guanine phosphoribosyltransferase (HPRT). The following primers were used: GAPDH: 5′-ggtgaaggtcggtgtgaacg-3′ and 5′-accatgtagttgaggtcaatgaagg-3′; Bcl-6: 5′-agacgcacagtgacaaacca-3′ and 5′-cgctccacaaatgttacagc-3′.

### Statistical analysis

Data were analyzed by a two-tailed Student's *t*-test using SPSS 11.0 software (IBM). The significance of the difference between the treatment groups was identified using a t-test. P-values of less than 0.05 were considered as statistically significant.

## Supporting Information

Figure S1 **Gating schemes for analysis of the percentage of Tfh cells.** Leukocytes from spleens, mesenteric LN, or livers after red blood cell lysis were stained with CD3-percpcy5.5, CD4-FITC, CXCR5-APC, and PD-1-PE antibodies. Gating strategy defining Tfh cells (R3). Data are representative of three independent experiments.
(TIF)

Figure S2 **The levels of SEA-specific IgG and IgG1 antibody in the sera from WT or ICOSL KO mice infected with or without** ***S. japonicum*** **.** The levels of SEA-specific IgG (A) and IgG1 (B) antibody in the sera from WT or ICOSL KO mice 8 weeks infected with or without *S. japonicum* were determined by ELISA. Data are expressed as the mean ± SD of 18 mice from three independent experiments, ***, P<0.001 (Student's *t*-test).
(TIF)

Figure S3 **The percentages of Th1, Th2, Th17 and Treg cells in ICOSL KO mice.** Mouse spleens, mesenteric LN, and livers from WT and ICOSL KO mice 8 weeks infected with or without *S. japonicum* were harvested and the cells were surface stained with CD3-APC and CD4-FITC, then followed by intra-cellular staining with IFN-gamma-PE, IL-4-PE, or IL-17-PE for Th1, Th2, or Th17 cells detection. Or cells were surface stained with CD4-FITC and CD25-APC, then followed by intra-cellular staining with Foxp3-PE after Fc blocking for Treg cells detection. The percentages of Th2 (A), Th17 (B), Th1 (C) in total CD3^+^T cells and Treg cells (D) in total CD4^+^ T cells from mouse spleens, mesenteric lymph nodes and livers. Cells were gated on the CD3^+^ population for analysis of Th2, Th17, Th1 cells, or gated on the CD4^+^ population for analysis of Treg cells. Data are expressed as the mean ± SD of 18 mice from three independent experiments, *, P<0.05, **, P<0.01, ***, P<0.001 (Student's *t*-test).
(TIF)

Figure S4 **The expression of CXCR5 in eGFP^+^ Tfh cells three weeks after transferring.** Hepatic lymphocytes from eGFP mice 8 weeks after *S. japonicum* infection (top) or ICOSL KO recipient mice 3 weeks after transferring of the eGFP^+^CXCR5^+^PD-1^+^CD4^+^ Tfh cells (bottom) were stained with CD3-percp-cy5.5, CD4-PE-Cy7, CXCR5-APC and PD-1-PE, or CXCR5-APC, PD-1-PE and isotype antibodies, respectively. Flow cytometric contour plot of CXCR5^+^PD-1^+^ cells (gated on CD3^+^CD4^+^ cells or eGFP^+^ cells). Data are representative of three independent experiments with 3 mice in each group.
(TIF)

Figure S5 **Macrophage-T cell conjugates in livers from** ***S. japonicum*** **-infected mice.** (A) Flow cytometry of CD4^+^CXCR5^+^F4/80^+^ doublets in the livers of *S. japonicum*-infected mice. Data are representative of three experiments with three mice in each group; (B) Size (forward scatter (FSC)) of CD4^+^CXCR5^+^F4/80^−^ singlet cells, CD4^−^CXCR5^−^F4/80^+^ singlet cells or CD4^+^CXCR5^+^F4/80^+^ doublets in the livers of *S. japonicum*-infected mice. Data are representative of three experiments.
(TIF)

Figure S6 **The expression of ICOSL on dendritic cells in normal and infected mice.** Splenocytes from normal and infected mice were stained with CD11c-FITC and ICOSL-PE antibodies. Flow cytometric histogram of ICOSL^+^ cells (gated on CD11c^+^ cells). Data are representative of three experiments with 3 mice in each group.
(TIF)

Figure S7 **The survival and migration of macrophages 7 days after transferring.** (A) Absolute numbers of eGFP macrophage in peritoneal cavity of the infected or uninfected control ICOSL KO recipient mice 1 or 7 days after adoptively transferring of the eGFP^+^ macrophage are shown. Data are expressed as the mean ± SD of 6 mice from two independent experiments with three mice in each group. ***, P<0.001 (Student's *t*-test); (B) Flow cytometric dot plots of eGFP^+^ cells from normal and infected mice recipients 7 days after adoptive transfer of the PBS, or eGFP^+^ macrophage. The numbers in the dot plots represent percentages of total cells.
(TIF)

Text S1 **Supporting text.** This file contains detailed methods, including measurement of Th1, Th2, Th17, and Treg cells by flow cytometry, plasticity of Tfh cells, and survival and migration of macrophages.
(DOC)
