# Supplementary figures and images for: Follicular Helper T Cells Promote Liver Pathology in Mice during Schistosoma japonicum Infection
Source: PLoS Pathog. 2014 May 1;10(5):e1004097. doi: 10.1371/journal.ppat.1004097 (PMC4006917; doi:10.1371/journal.ppat.1004097)

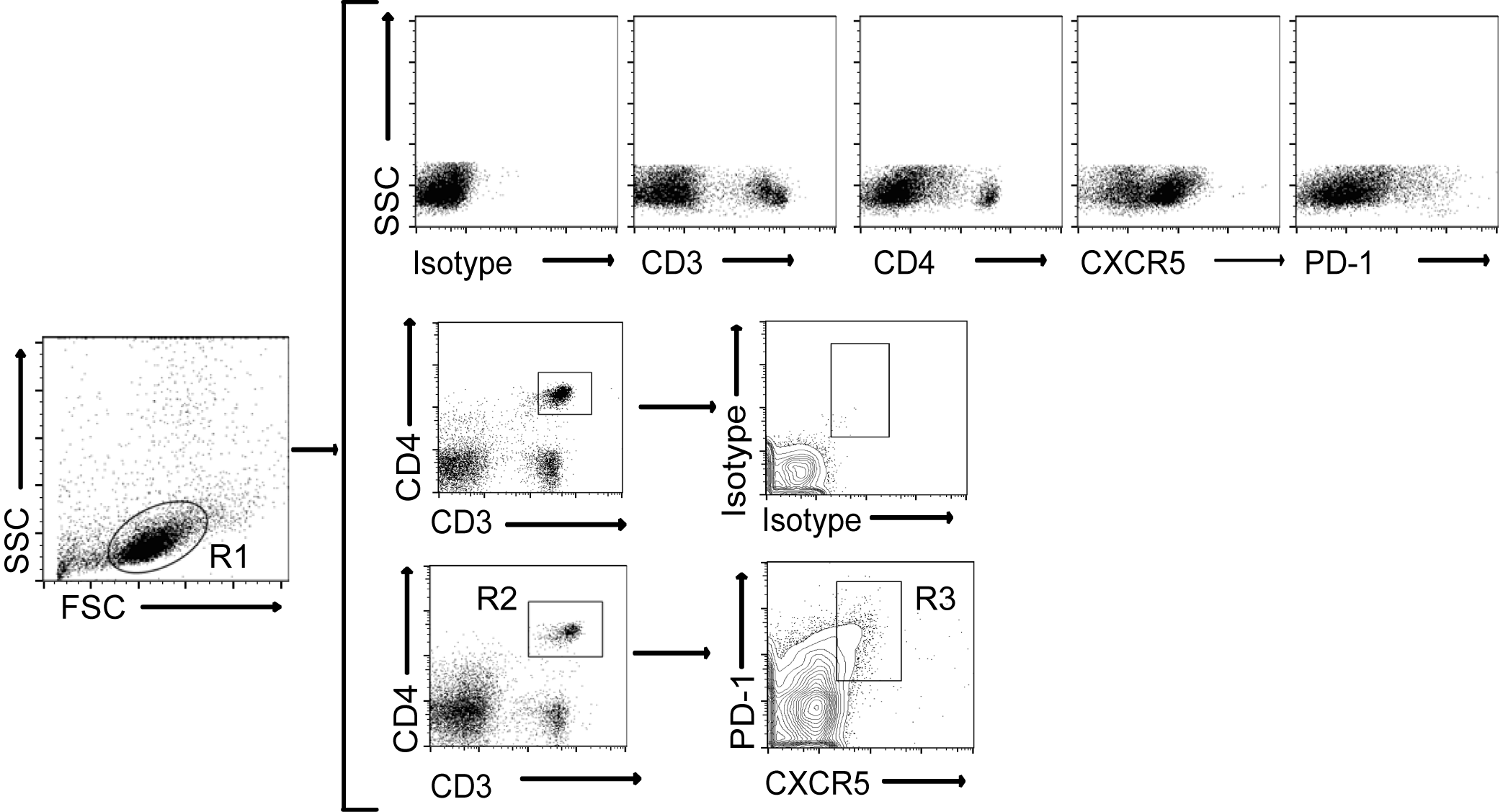

Supplement: Figure S1 — Gating schemes for analysis of the percentage of Tfh cells. Leukocytes from spleens, mesenteric LN, or livers after red blood cell lysis were stained with CD3-percpcy5.5, CD4-FITC, CXCR5-APC, and PD-1-PE antibodies. Gating strategy defining Tfh cells (R3). Data are representative of three independent experiments. (TIF) [file ppat.1004097.s001.tif]

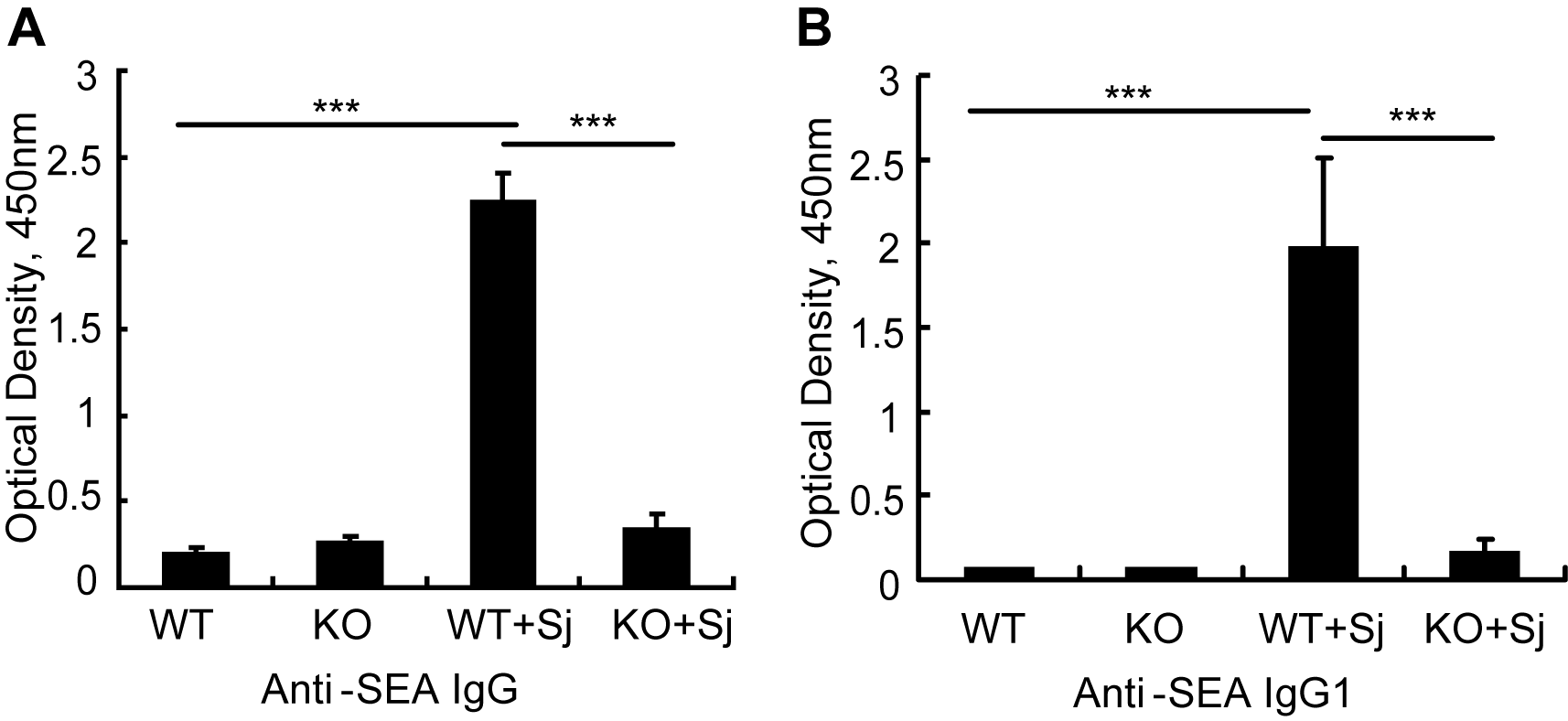

Supplement: Figure S2 — The levels of SEA-specific IgG and IgG1 antibody in the sera from WT or ICOSL KO mice infected with or without S. japonicum . The levels of SEA-specific IgG (A) and IgG1 (B) antibody in the sera from WT or ICOSL KO mice 8 weeks infected with or without S. japonicum were determined by ELISA. Data are expressed as the mean ± SD of 18 mice from three independent experiments, ***, P<0.001 (Student's t-test). (TIF) [file ppat.1004097.s002.tif]

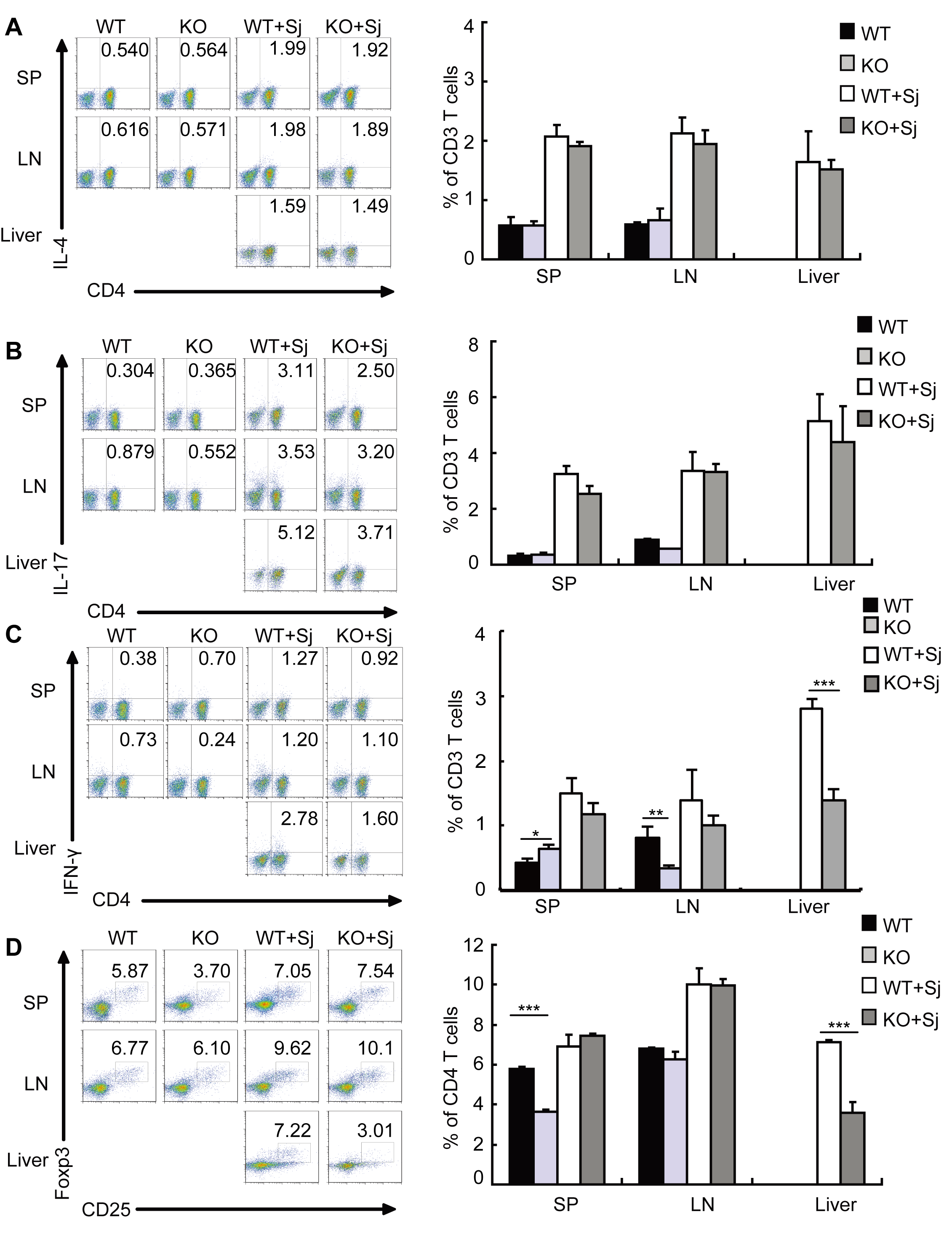

Supplement: Figure S3 — The percentages of Th1, Th2, Th17 and Treg cells in ICOSL KO mice. Mouse spleens, mesenteric LN, and livers from WT and ICOSL KO mice 8 weeks infected with or without S. japonicum were harvested and the cells were surface stained with CD3-APC and CD4-FITC, then followed by intra-cellular staining with IFN-gamma-PE, IL-4-PE, or IL-17-PE for Th1, Th2, or Th17 cells detection. Or cells were surface stained with CD4-FITC and CD25-APC, then followed by intra-cellular staining with Foxp3-PE after Fc blocking for Treg cells detection. The percentages of Th2 (A), Th17 (B), Th1 (C) in total CD3+T cells and Treg cells (D) in total CD4+ T cells from mouse spleens, mesenteric lymph nodes and livers. Cells were gated on the CD3+ population for analysis of Th2, Th17, Th1 cells, or gated on the CD4+ population for analysis of Treg cells. Data are expressed as the mean ± SD of 18 mice from three independent experiments, *, P<0.05, **, P<0.01, ***, P<0.001 (Student's t-test). (TIF) [file ppat.1004097.s003.tif]

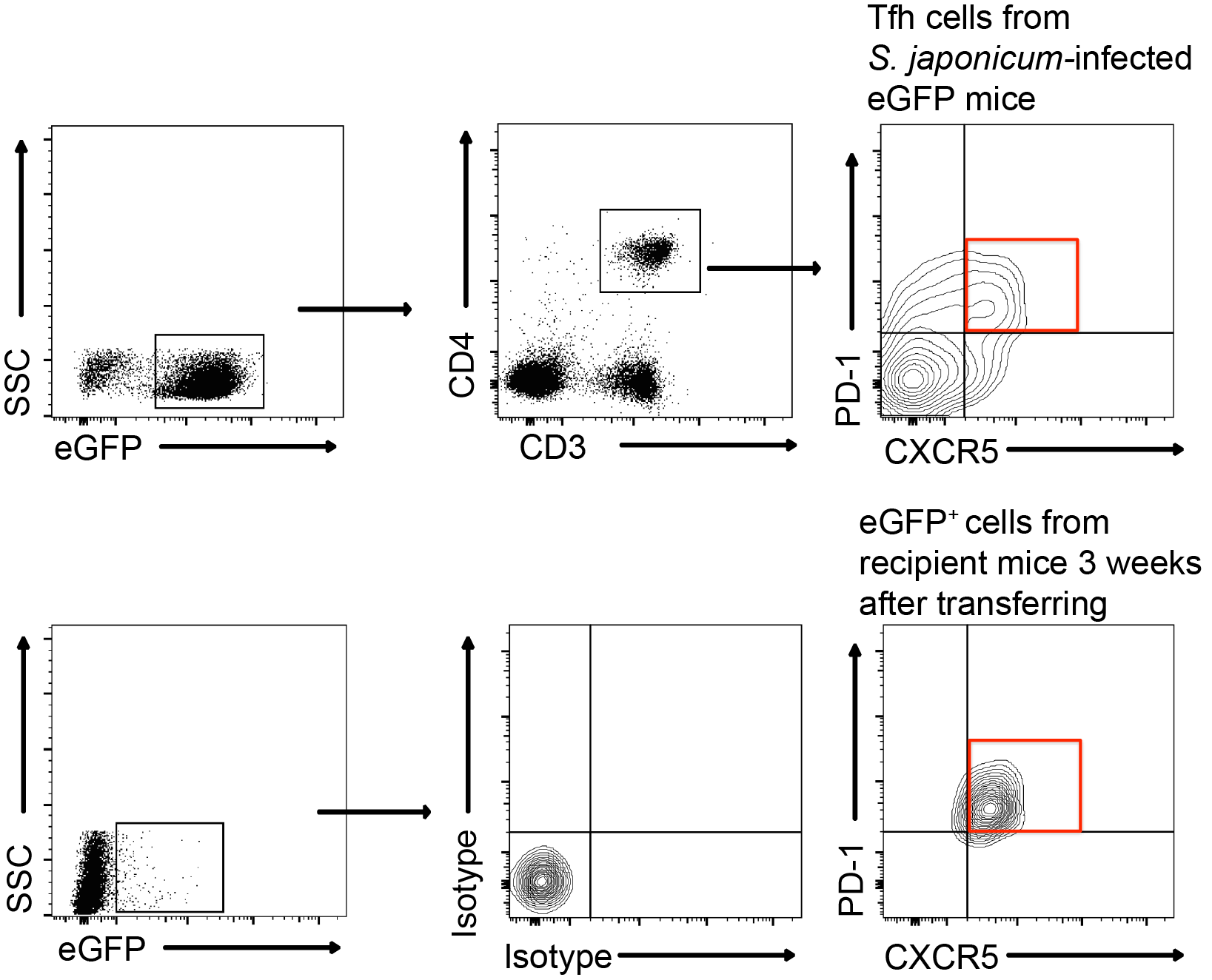

Supplement: Figure S4 — The expression of CXCR5 in eGFP+ Tfh cells three weeks after transferring. Hepatic lymphocytes from eGFP mice 8 weeks after S. japonicum infection (top) or ICOSL KO recipient mice 3 weeks after transferring of the eGFP+CXCR5+PD-1+CD4+ Tfh cells (bottom) were stained with CD3-percp-cy5.5, CD4-PE-Cy7, CXCR5-APC and PD-1-PE, or CXCR5-APC, PD-1-PE and isotype antibodies, respectively. Flow cytometric contour plot of CXCR5+PD-1+ cells (gated on CD3+CD4+ cells or eGFP+ cells). Data are representative of three independent experiments with 3 mice in each group. (TIF) [file ppat.1004097.s004.tif]

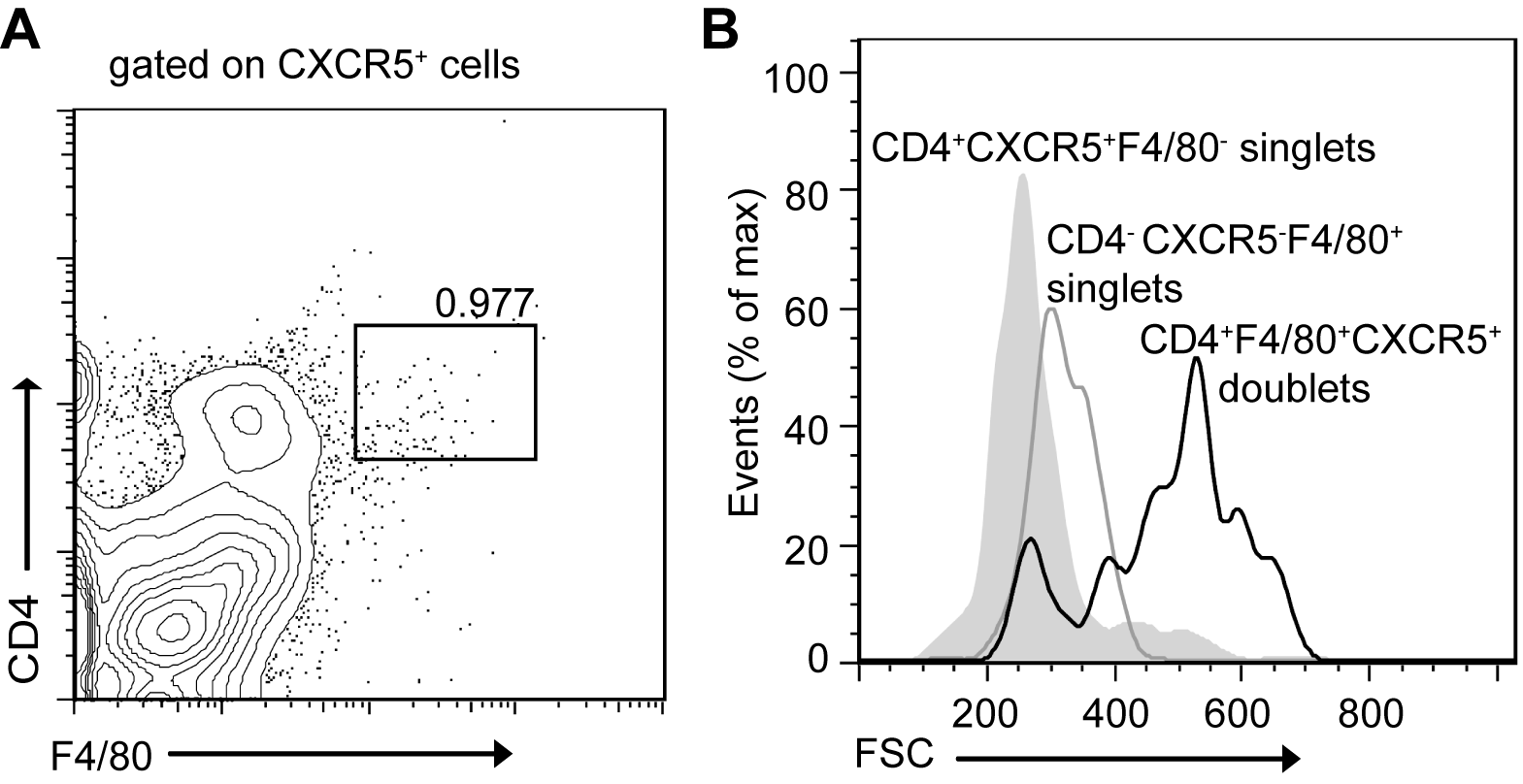

Supplement: Figure S5 — Macrophage-T cell conjugates in livers from S. japonicum -infected mice. (A) Flow cytometry of CD4+CXCR5+F4/80+ doublets in the livers of S. japonicum-infected mice. Data are representative of three experiments with three mice in each group; (B) Size (forward scatter (FSC)) of CD4+CXCR5+F4/80− singlet cells, CD4−CXCR5−F4/80+ singlet cells or CD4+CXCR5+F4/80+ doublets in the livers of S. japonicum-infected mice. Data are representative of three experiments. (TIF) [file ppat.1004097.s005.tif]

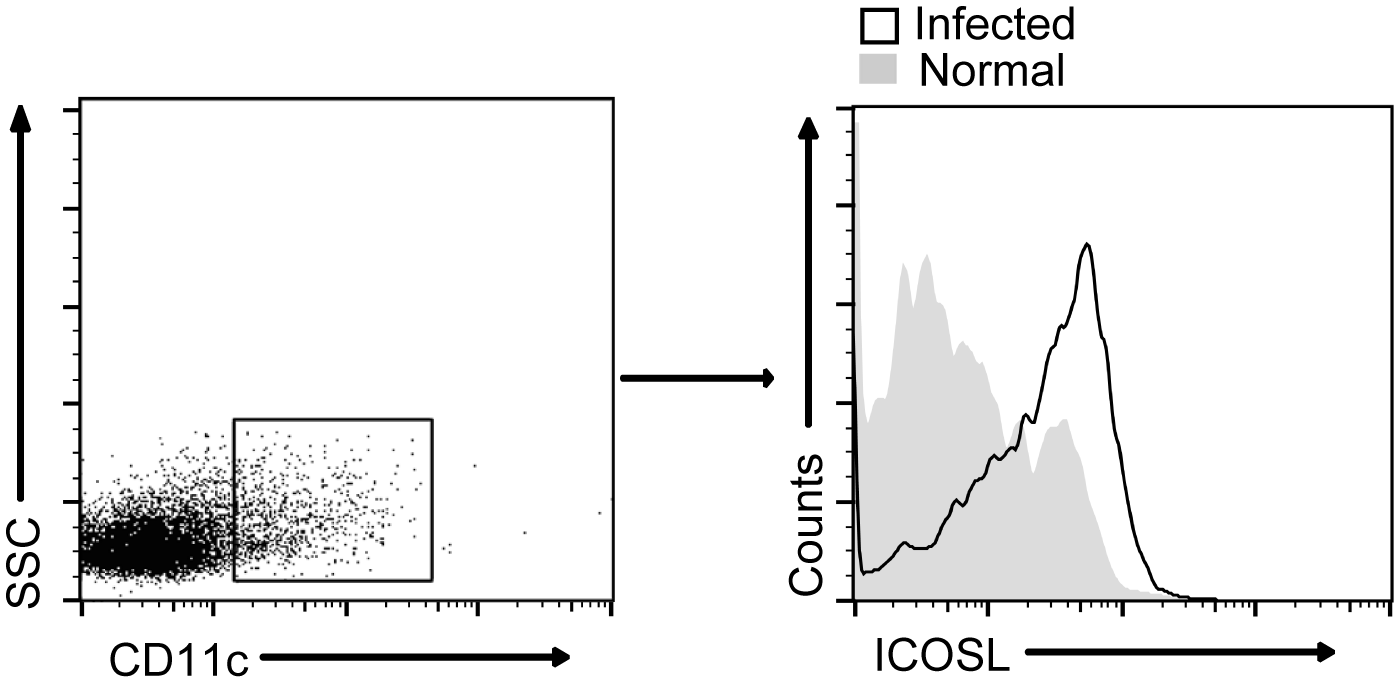

Supplement: Figure S6 — The expression of ICOSL on dendritic cells in normal and infected mice. Splenocytes from normal and infected mice were stained with CD11c-FITC and ICOSL-PE antibodies. Flow cytometric histogram of ICOSL+ cells (gated on CD11c+ cells). Data are representative of three experiments with 3 mice in each group. (TIF) [file ppat.1004097.s006.tif]

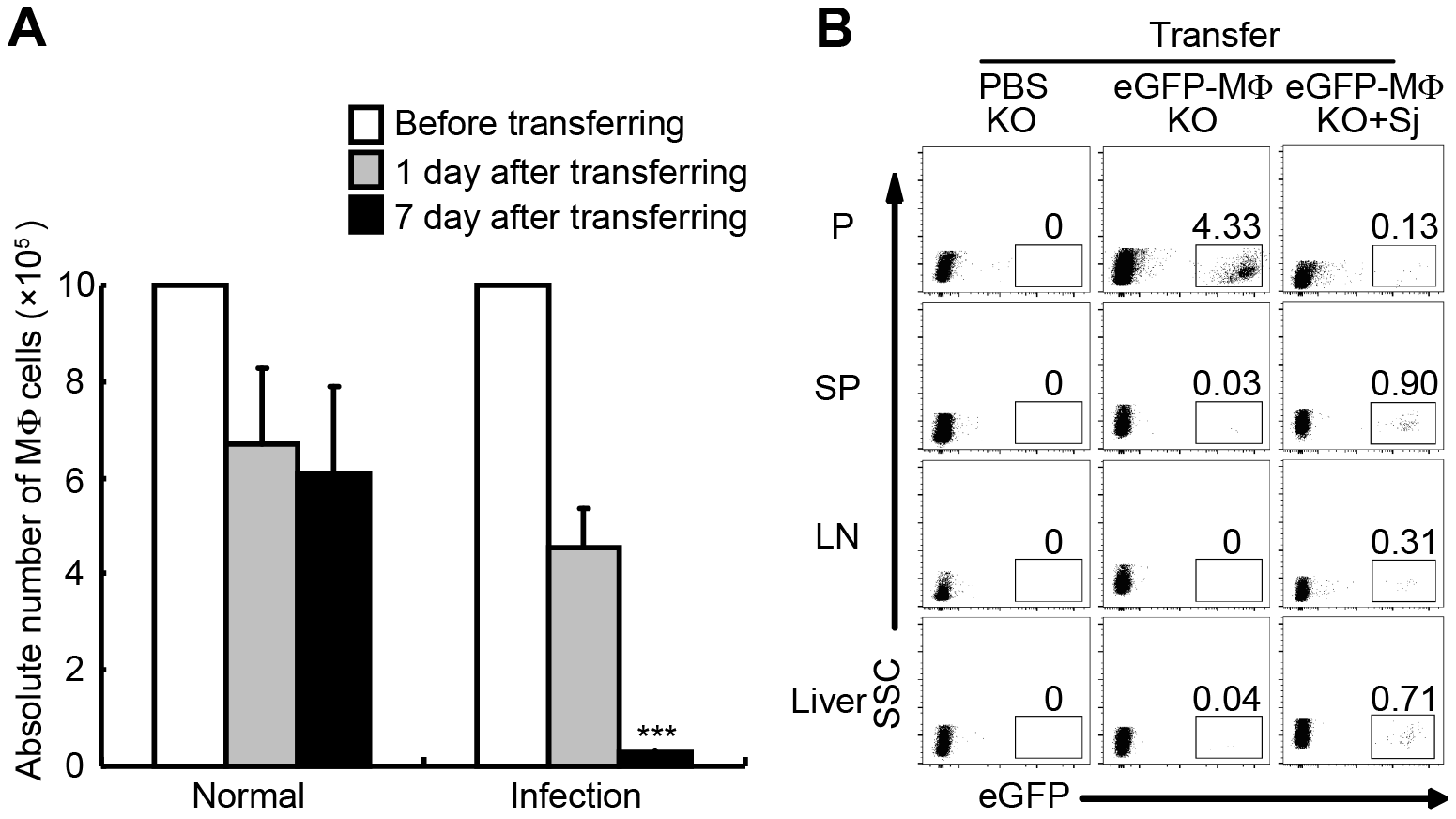

Supplement: Figure S7 — The survival and migration of macrophages 7 days after transferring. (A) Absolute numbers of eGFP macrophage in peritoneal cavity of the infected or uninfected control ICOSL KO recipient mice 1 or 7 days after adoptively transferring of the eGFP+ macrophage are shown. Data are expressed as the mean ± SD of 6 mice from two independent experiments with three mice in each group. ***, P<0.001 (Student's t-test); (B) Flow cytometric dot plots of eGFP+ cells from normal and infected mice recipients 7 days after adoptive transfer of the PBS, or eGFP+ macrophage. The numbers in the dot plots represent percentages of total cells. (TIF) [file ppat.1004097.s007.tif]
