# Supplementary material for: Follicular Helper T Cells Promote Liver Pathology in Mice during Schistosoma japonicum Infection
Source: PLoS Pathog. 2014 May 1;10(5):e1004097. doi: 10.1371/journal.ppat.1004097 (PMC4006917; doi:10.1371/journal.ppat.1004097)
Supplement: Text S1 — Supporting text. This file contains detailed methods, including measurement of Th1, Th2, Th17, and Treg cells by flow cytometry, plasticity of Tfh cells, and survival and migration of macrophages. (DOC) [file ppat.1004097.s008.doc]

**Supplementary Information**

**Materials and Methods**

**Flow cytometry**

For surface staining, 2 × 10^6^ cells per 100 μl were incubated for 45 min at 4 ℃ with the following labeled monoclonal antibodies: CD3-percp-cy5.5 (eBioscience), CD4-PE-Cy7 (eBioscience), CXCR5-APC (BD Pharmingen), PD-1-PE (eBioscience), CD4-FITC (eBioscience), CD3e-APC (eBioscience), CD19-FITC (eBioscience), CD11c-FITC (eBioscience), F4/80-FITC (eBioscience), ICOSL-PE (eBioscience), and isotype antibodies. For intracellular cytokine staining, cells were isolated as described above and were stimulated for 4 h at 37℃, in a humidified atmosphere of 10% CO_2_ , in culture medium containing PMA (25ng/ml; Sigma), ionomycin (1 μg/ml; Sigma) and monensin (Golgi Stop; 1 μg/ml; BD Biosciences). After staining of surface markers, cells were fixed and made permeable with Cytofix/Cytoperm and Perm/Wash buffer according to the manufacturer’s instructions (BD Biosciences), then labeled with the following monoclonal antibodies: IFN-γ-PE (eBioscience), IL-4-PE (eBioscience) and IL-17-PE (eBioscience). Cells were incubated for 20 min at 4℃ and washed twice in Perm/Wash before analysis

To evaluate the percentages of CD4^+^CD25^+^Foxp3^+^ Treg cells, a total of 2 × 10**^6^** cells were stained with CD4-FITC (eBioscience) and CD25-APC (eBioscience). The cells were subsequently permeabilized with cold Fix/Perm Buffer, and Fc receptors of cells were blocked with anti-mouse CD16/32 (eBioscience) for 15 min. The Foxp3-PE (eBioscience) or PE-labeled rRat IgG2a isotype control antibody (eBioscience) was then added based on the manufacturer’s recommendations. The cells were then washed twice in wash buffer before analysis.

**Adoptive transfer experiments**

Spleen and mesenteric LN cells from eGFP C57BL/6J mice 8 weeks after infection with *S*. *japonicum* were pooled, and CD4^+^ T cells were presorted by using a CD4^+^ T cell negative-isolation kit (Miltenyi Biotec). The eGFP^+^CD4^+^ T cells were stained with CXCR5-APC, and PD-1-PE antibodies. The eGFP^+^CXCR5^high^PD-1^high^ Tfh cells were FACS purified by using a FACSAria cell sorter. FACS-sorted Tfh cells were resuspended in PBS and injected intraperitoneally (ip) into the ICOSL^-/-^ mice 5 weeks after *S*. *japonicum* infection (3 × 10^6^ cells/mouse). Mice were sacrificed 3 weeks after transfer to investigate the expression of CXCR5 and PD-1 on the transferred eGFP^+^ cells.

The eGFP^+^ peritoneal macrophages (eGFP^+^PMs) from eGFP mice were prepared as described, resuspended in PBS and injected ip into the normal mice or mice 8 weeks after *S*. *japonicum* infection (1 × 10^6^ cells/mouse). Mice were sacrificed 1 or 7 days after transferring to investigate the survival and migration of macrophage *in vivo*.
